# Evaluating heart rate variability with 10 second multichannel electrocardiograms in a large population-based sample

**DOI:** 10.3389/fcvm.2023.1144191

**Published:** 2023-05-12

**Authors:** Elischa Krause, Marcus Vollmer, Katharina Wittfeld, Antoine Weihs, Stefan Frenzel, Marcus Dörr, Lars Kaderali, Stephan B. Felix, Beate Stubbe, Ralf Ewert, Henry Völzke, Hans J. Grabe

**Affiliations:** ^1^ Department of Psychiatry and Psychotherapy, University Medicine Greifswald, Greifswald, Germany; ^2^ Institute of Bioinformatics, University Medicine Greifswald, Greifswald, Germany; ^3^ German Centre for Cardiovascular Research (DZHK), Partner Site Greifswald, Greifswald, Germany; ^4^ German Centre for Neurodegenerative Diseases (DZNE), Partner Site Rostock/Greifswald, Greifswald, Germany; ^5^ Department of Internal Medicine B, University Medicine Greifswald, Greifswald, Germany; ^6^ Institute for Community Medicine, University Medicine Greifswald, Greifswald, Germany

**Keywords:** heart rate variability (HRV), ECG, RMSSD, biomarker, PHQ-9, depression

## Abstract

**Introduction:**

Heart rate variability (HRV), defined as the variability of consecutive heart beats, is an important biomarker for dysregulations of the autonomic nervous system (ANS) and is associated with the development, course, and outcome of a variety of mental and physical health problems. While guidelines recommend using 5 min electrocardiograms (ECG), recent studies showed that 10 s might be sufficient for deriving vagal-mediated HRV. However, the validity and applicability of this approach for risk prediction in epidemiological studies is currently unclear to be used.

**Methods:**

This study evaluates vagal-mediated HRV with ultra-short HRV (usHRV) based on 10 s multichannel ECG recordings of *N* = 4,245 and *N* = 2,392 participants of the Study of Health in Pomerania (SHIP) from two waves of the SHIP-TREND cohort, additionally divided into a healthy and health-impaired subgroup. Association of usHRV with HRV derived from long-term ECG recordings (polysomnography: 5 min before falling asleep [*N* = 1,041]; orthostatic testing: 5 min of rest before probing an orthostatic reaction [*N* = 1,676]) and their validity with respect to demographic variables and depressive symptoms were investigated.

**Results:**

High correlations (*r* = .52–.75) were revealed between usHRV and HRV. While controlling for covariates, usHRV was the strongest predictor for HRV. Furthermore, the associations of usHRV and HRV with age, sex, obesity, and depressive symptoms were similar.

**Conclusion:**

This study provides evidence that usHRV derived from 10 s ECG might function as a proxy of vagal-mediated HRV with similar characteristics. This allows the investigation of ANS dysregulation with ECGs that are routinely performed in epidemiological studies to identify protective and risk factors for various mental and physical health problems.

## 1. Introduction

The dysregulation of the autonomic nervous system (ANS) has gained broad scientific interest over the last decades as it is associated with the development, course, and outcome of a variety of health problems (1–6). Besides, the ANS is crucial for flexible adaptation of an individual to changing environmental demands by keeping its two major branches—the sympathetic and parasympathetic nervous system—in dynamic balance. However, when these branches are becoming static imbalanced, for example, due to chronic stress, the ability of selecting and orchestrating an appropriate response is reduced or even vanished, facilitating the emergence of serious mental and physical health problems (7–10).

In this context, heart rate variability (HRV), defined as the variability of the duration of consecutive heart beats, has been proven to be a reliable non-invasive biomarker of ANS functioning (11, 12), which is stable over longer periods of time (13, 14). HRV has been studied extensively and a broad field of research has found higher HRV to be associated, for example, with better hypothalamic-adrenal-pituitary axis function, reduced inflammation, better glucose regulation (15–19), and also with better higher functions for adaption and goal directed behavior (20–25). In contrast, lower HRV is associated with higher risk for stroke, cardiovascular disease (CVD), and all-cause mortality (4, 15) as well as with mental disorders, like, among others, depression (6, 26, 27).

Following HRV guidelines, the gold standard recommends a minimum of 5 min electrocardiogram (ECG) recording while the participant is at rest (28, 29). There are many different HRV parameters based on time and frequency domain methods, whereas high frequency HRV (HF-HRV) and the root mean square of successive differences (RMSSD) are widely used as indices of parasympathetic activity/vagal tone (11, 29, 30). However, the RMSSD is assumed to provide a better assessment of vagal tone as it is mostly unaffected from respiratory (31).

Even though HRV is a powerful tool, it is rarely applied in clinical settings or epidemiological studies as the methodology in line with the guidelines is among other things time consuming and therefore its practicability is limited. Therefore, previous studies had challenged the gold standard by demonstrating that ultra-short term HRV, based on much shorter ECGs (less than 1 min), might be a suitable and valid surrogate, especially for vagal-mediated HRV, i.e., RMSSD (32). Some studies identified 30 s or even 20 s ECGs as the minimal length for reliable estimating RMSSD (33–35), while there is evidence that a 10 s ECG segment might be enough (35). Munoz et al. (36) compared several HRV parameters derived from 4 to 5 min ECGs with segments of different lengths in a large population-based study and demonstrated that a single 10 s ECG segment was suitable for estimating a valid RMSSD. This is in accordance with findings from studies with smaller sample sizes, consisting of healthy individuals (37–40) and patients with diabetes mellitus (41) and dyslipidemia (42). While these studies used a short segment of the same ECG for analysis, similar results were found with separate ECGs of 10 s and 5 min length (43).

Despite the presented evidence for the validity of HRV derived from 10 s ECG (usHRV), there are some questions and concerns. First, as only two out of eleven studies mentioned above had a sample size larger than 100 (33, 36), some statistical analyses may have been affected by a lack of statistical power. Secondly, nine out of eleven studies included only healthy individuals, reducing the generalization of their results to investigate physical and especially mental health problems. Thus, the question of applicability and validity of usHRV in population-based samples cannot be fully answered yet. Thirdly, nine out of eleven presented studies estimated and compared HRV parameters from different segments of the same ECG, mainly focusing on the methodical aspect of obtaining ultra-short term HRV. Only Hamilton et al. (38) splitted their study sample for a quasi-comparison, while Boos et al. (43) used separate ECGs. Thus, to our knowledge, there is no study comparing 10 s HRV with HRV estimated from longer ECG that has been recorded at different time points to evaluate as well as challenge the validity and robustness of usHRV. Fourthly, and most importantly, there are only a few studies that investigated associations of 10 s HRV with physical and mental health problems (44–46), but the results were not validated with the gold standard or at least with HRV derived from longer ECG recordings at rest.

Taken together, there is some evidence for using usHRV estimated from 10 s ECG, however, the validity and applicability in epidemiological studies has not yet been systematically investigated and therefore remains to be proven. Moreover, 10 s 12-channel ECGs are very often performed in population-based cohorts (e.g., in all waves of the SHIP cohorts), but longer ECG recordings at rest are rare, especially in a longitudinal manner. Therefore, this study explores the associations between RMSSD derived from standard 12-channel 10 s ECG with segments of different lengths of a one-night polysomnography (PSG) and the 5 min resting period before probing an orthostatic reaction. Moreover, as associations between usHRV and HRV do not necessarily answer the question of applicability and validity of usHRV, we additionally evaluate known associations of HRV with typical demographic variables, like age and sex, and the obesity index waist-to-height-ratio (WHtR) (47–50) across different ECG origins. Furthermore, as depression is highly prevalent in general population (51) and has found to be linked with CVD (52), we explore the association of HRV with depressive symptoms from an epidemiological point of view. Additionally, the samples were divided into subgroups of healthy and health issues, based on self-reported health problems, in order to differentiate the possible effects of HRV alterations due to health impairments.

In general, these analyses are based on the assumption that vagal mediated HRV is stable over longer periods of time and therefore has a trait-like character (13, 14) that can be derived at any time point of rest. Thus, we expect to find substantial support for our assumption that usHRV at rest is a proxy for vagal-mediated HRV, irrespective of time differences between ECG recordings. Moreover, as 10 s of ECG recording is really short and therefore each detected heartbeat contributes a significant amount of information, either to the direction of higher or lower HRV, we used a new self-developed multichannel approach for increasing the reliability of usHRV. Nevertheless, usHRV is expected to be a proxy and therefore to be less accurate, resulting into potential level differences of RMSSD values, biases, and larger variation.

## 2. Materials and methods

### 2.1. Study population

The study samples are taken from the second cohort of the population-based Study of Health in Pomerania (SHIP-TREND) (53), which is a general population-based cohort in Northeastern Germany. The SHIP-TREND baseline sample (SHIP-TREND-0: 2008–2012; *N* = 4,420) was randomly drawn from local registries in the north-east of Mecklenburg-Western Pomerania in Germany. A subgroup of the participants volunteered for an overnight PSG [28.6%; *N* = 1,264; for details see Stubbe et al. (54)]. In the first follow-up study wave (SHIP-TREND-1: 2016–2019; *N* = 2,507) the majority participated in another voluntary examination module of an orthostatic testing which included ECG recording (71.56%; *N* = 1,794). Nearly all participants of the baseline and the follow-up wave underwent a short standard 10 s electrocardiographic examination (SHIP-TREND-0: 99.5%, *N* = 4,396; SHIP-TREND-1: 99.4%, *N* = 2,493). A more in-depth analysis of the cohort can be found by Völzke et al. (53) and Stubbe et al. (54).

The SHIP study was performed by trained and certified staff. Data acquisition was performed according to the Declaration of Helsinki and all participants gave written informed consent. The study protocols were approved by the institutional review board of the University Greifswald, Germany.

### 2.2. Data assessment

A computer-assisted face-to-face interview and self-report questionnaires were used to assess, among others, data on CVD (e.g., previous heart attacks, atrial fibrillation, and pacemaker) and depressive symptoms (55, 56). Moreover, all subjects underwent medical examinations in order to assess, among others, the WHtR as an estimate for obesity (57).

### 2.3. Electrocardiography

#### 2.3.1. 12-lead ECG

The 12-lead ECGs in SHIP-TREND-0 and SHIP-TREND-1 were performed following standard clinical procedures (58). Specifically, participants were placed in supine position in a quiet room (temperature 23°C ± 2°C) and asked to relax. Before the electrodes were placed on all limbs and on the surface of the chest, the skin areas were cleaned for optimal signal quality. Then, a 10 s ECG was recorded using a CardioPerfect-ECG-System (WelchAllyn Inc., Auburn, NY, USA) which simultaneously records a 12-lead ECG (leads: Einthoven I, II & III; Goldberger aVR, aVL & aVF and Wilson V1–V6) with a sampling rate of 500 Hz. During the whole examination, the examiner tried to establish a calm and relaxed atmosphere.

#### 2.3.2. Polysomnography

One-night PSG in SHIP-TREND-0 was performed as described by Stubbe et al. (54) according to AASM standards (59) using ALICE 5 PSG devices (Philips Respironics, Eindhoven, Netherlands). All sensors were placed carefully, and, among others, body position and a one channel ECG (Einthoven II) was recorded with a sampling rate of 200 Hz. Moreover, all signals were checked and, if necessary, calibrated to ensure optimal signal quality. Participants could choose their own bedtime and wake-up time, whereas a total in bedtime of 8 h was requested.

#### 2.3.3. Orthostatic testing

Participants of SHIP-TREND-1 were instructed about the procedure and placed in supine position in a quiet room (temperature 22–24°C). Then, three ECG electrodes were placed on cleaned skin areas (right chest, left and right abdomen), allowing to record a 6-channel ECG (leads: Einthoven I, II and III; Goldberger aVR, aVL & aVF) using the SOMNOtouch NIBP device (SOMNOmedics GmbH, Randersacker, Germany) with a sampling rate of 256 Hz. After the signal quality was checked and the recording started, participants had to rest for four minutes. Then, after measuring blood pressure (2 min), participants had to rest again (5 min segment for HRV estimation; total rest time 11 min) before they had to change their position from supine into stand for three minutes (probing orthostatic reaction).

### 2.4. Patient health questionnaire—PHQ-9

The PHQ-9 is a module of the Patient Health Questionnaire and is a well-established self-report screening instrument for symptoms of depression with a high reliability (.86–.89) and validity (55, 60). It contains nine items, scoring the corresponding DSM-IV criterion of depression within the last two weeks from 0 (“not at all”) to 3 (“almost every day”), which can be summarized into a depression symptom severity score (range 0–27).

### 2.5. Data preparation

#### 2.5.1. HRV

HRV was estimated with a newly developed in-house pipeline for analyzing multichannel HRV in MATLAB (ver. R2019a; MathWorks, Inc., Natick, Massachusetts, USA) based on the HRVTool toolbox (61).

First, for each lead separately, all QRS-complexes (defined as 60 ms around the peak of an R wave) detected with the HRVTool were averaged to create a subject and lead specific QRS-template. Then, the QRS-template was correlated with each detected QRS-complex to exclude falsely detected or abnormal heart beats, such as ectopic beats, extrasystole and erratic sinus beats. Additionally, in order to check for inverted leads in multichannel ECG recordings, a general cohort, ECG-measurement and lead specific QRS-template was created based on the corresponding subject specific QRS-templates, and then, the distribution of its correlation with each subject specific QRS-template was estimated. If the subject specific correlation was below two standard deviations (SD) of the median of this distribution, the ECG signal was inverted, and the described procedure above repeated to compare both correlations and to select the best fitting one. Afterwards, the timings of the peaks of the remaining R waves were determined by using sample rate free spline interpolation (spline; build-in function in MATLAB). Adjacent RR intervals were excluded, wherever a heartbeat was classified as non-normal. Then, heart rate (HR) and the HRV parameter RMSSD was calculated for each subject and lead. Additionally, in the case of a multi-channel ECG recording, the subject specific median and SD of the calculated parameters were estimated and only those values within one SD of the median were averaged. Moreover, ECGs with abnormal values (i.e., RMSSD, HR, numbers of RR intervals) were visually inspected and, if necessary, excluded.

#### 2.5.2. 12-lead ECG

To improve the validity of usHRV derived from 10 s ECG, additional quality control steps were applied. Thus, as the mean correlation of the cohort specific QRS-template with the subject specific QRS-templates was too low for five out of 12 leads (correlation less than .7—explaining less than ∼50% variance), only the leads Einthoven I and II, Goldberger aVR and Wilson V3-V6 were used for estimating the usHRV parameter RMSSD in this study. Moreover, only those values with the highest number of the same count of valid RR-intervals (RRcnt), with a minimum criterion of five, were averaged and only RMSSD-values below 250 ms with at least two valid leads were used for further statistical analyses (drop-out SHIP-TREND-0: *N* = 29; SHIP-TREND-1: *N* = 60).

#### 2.5.3. Polysomnography

Two time windows were used to estimate HRV from PSG recording: *HRV*: A time window of 5 min was used where the participant was lying and still awake (mean time of rest before the 5 min segment: *M*[SD] = 28.56[26.37] min. Moreover, RMSSD-values were only included when they were below five SD of the mean (RMSSD_range_: 0–173.8) and their count of valid RR-intervals were not extreme outliers (RRcnt_range_: 78–561). *Polysomnography-HRV (PSG-HRV)*: The complete ECG recording during the polysomnography was used for estimating long-term HRV. RMSSD-values were only included, with a time range of 5–11 h and at least 5,000 valid RR-intervals.

#### 2.5.4. Orthostatic testing

An ECG segment of 5 min of rest before probing the orthostatic reaction was used for estimating HRV. For statistical analyses, RMSSD-values were included if they were below five SD of the mean (RMSSD_range_: 0–180.5), their count of valid RR-intervals were not extreme outlier (RRcnt_range_: 671–564) and had at least two valid leads.

#### 2.5.5. Segmentation

Additionally, 5 min ECG data from the PSG, where participants were awake, and the 5 min ECG data of rest before probing orthostatic reactions were segmented in 30 10 s segments and HRV were estimated for correlation and univariate analyses.

#### 2.5.6. Samples

**Figure 1:**
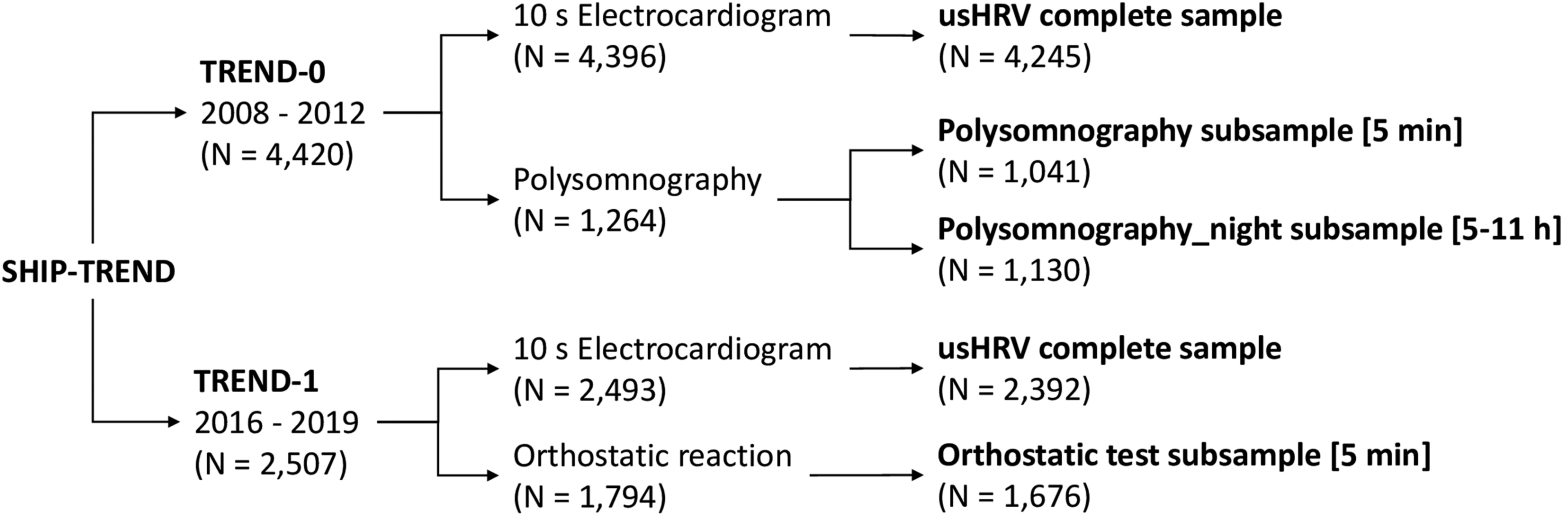
Flowchart of the generated five data (sub-)samples based on the available ECG data in SHIP-TREND-0 and SHIP-TREND-1.

ECG data was used from two waves of the SHIP-TREND cohort (SHIP-TREND-0: *N* = 4,420; SHIP-TREND-1: *N* = 2,507), where a 10 s ECG was available in both waves for nearly all participants (SHIP-TREND-0: *N* = 4,396; SHIP-TREND-1: *N* = 2,493). In addition, PSG recordings in SHIP-TREND-0 (*N* = 1,264) and probing of orthostatic reactions in SHIP-TREND-1 (*N* = 1,794) were analyzed. Based on these data sets, five data samples were created (see Table 1 and Figure 1) and participants with specific heart problems (i.e., pacemaker or atrial fibrillation) or who were pregnant during examination, were excluded. Three samples contained both long and ultrashort recording (SHIP-TREND-0: Polysomnography subsample [5 min] (PSG subsample): HRV and usHRV: *N* = 1,041; Polysomnography_night subsample [5–11 h] (PSG_night subsample): PSG-HRV and usHRV: *N* = 1,130; SHIP-TREND-1: Orthostatic test subsample (OT subsample): HRV and usHRV: *N* = 1,676) and two samples contained 10 s ECGs only (usHRV from SHIP-TREND-0: *N* = 4,245; usHRV from SHIP-TREND-1: *N* = 2,392).

**Table 1:** Sample characteristics.

Wave	SHIP-TREND-0						SHIP-TREND-1					
Sample	Polysomnography subsample [5 min]			usHRV complete sample [10 s]			Orthostatic test subsample [5 min]			usHRV complete sample [10 s]		
Subgroup	Complete	Healthy	Health issues	Complete	Healthy	Health issues	Complete	Healthy	Health issues	Complete	Healthy	Health issues
*N*	1,041	930	111	4,245	3,623	625	1,676	1,317	359	2,392	1,854	541
Female sex (%)	487 (46.8)	446 (48.0)	41 (36.9)	2,215 (52.2)	1,951 (53.9)	267 (42.7)	848 (50.6)	730 (55.4)	118 (32.9)	1,262 (52.8)	1,060 (57.2)	205 (37.9)
*p*-value			.026^b^			<.001^b^			<.001^b^			<.001^b^
Age [SD]	52.69 [13.76]	51.92 [13.61]	59.17 [13.34]	51.46 [15.25]	50.25 [15.04]	56.78 [15.42]	56.86 [13.11]	55.16 [12.70]	62.91 [12.74]	56.76 [13.71]	54.54 [13.12]	63.93 [13.21]
*p*-value			<.001^a^			<.001^a^			<.001^a^			<.001^a^
WHtR [SD]^c^	0.54 [0.08]	0.54 [0.08]	0.55 [0.08]	0.54 [0.08]	0.53 [0.08]	0.55 [0.08]	0.57 [0.08]	0.56 [0.08]	0.60 [0.08]	0.56 [0.08]	0.56 [0.08]	0.60 [0.08]
*p*-value			.076^a^			<.001^a^			<.001^a^			<.001^a^
BMI [SD]^d^	28.44 [4.87]	28.40 [4.87]	28.76 [4.83]	28.03 [5.18]	27.93 [5.20]	28.60 [5.07]	28.09 [4.87]	27.71 [4.74]	29.44 [5.11]	27.96 [4.94]	27.57 [4.86]	29.24 [4.98]
			.452			.002			<.001			<.001
Weight in KG [SD]^d^	83.08 [15.78]	83.00 [15.80]	83.76 [15.71]	80.97 [16.76]	80.62 [16.77]	83.01 [16.54]	81.85 [16.44]	80.50 [16.10]	86.67 [16.73]	81.11 [16.67]	79.78 [16.40]	85.41 [16.85]
			.632			<.001			<.001			<.001
Height in cm [SD]^c^	170.84 [9.18]	170.88 [9.19]	170.51 [9.11]	169.82 [9.40]	169.75 [9.43]	170.22 [9.22]	170.50 [9.25]	170.22 [9.27]	171.50 [9.12]	170.08 [9.46]	169.88 [9.50]	170.70 [9.31]
			.689			.240			.018			.068
HRV - RMSSD [SD]	34.67 [23.60]	33.96 [21.81]	40.65 [34.73]				29.08 [23.67]	27.58 [19.22]	34.39 [34.69]			
*p*-value			.049^a^						<.001^a^			
usHRV - RMSSD [SD]	28.47 [25.79]	27.70 [23.23]	34.93 [41.08]	29.51 [27.01]	29.13 [25.50]	31.68 [34.35]	28.46 [30.27]	26.83 [23.94]	34.25 [45.79]	28.67 [30.18]	27.25 [24.27]	33.38 [44.05]
*p*-value			.071^a^			.077^a^			.003^a^			.002^a^
HR 5 min [SD]	66.95 [9.39]	64.98 [9.29]	65.32 [10.54]				67.71 [9.79]	68.10 [9.54]	66.32 [10.52]			
*p*-value			.083^a^						.003^a^			
HR 10 s [SD]	64.83 [9.41]	64.98 [9.29]	63.59 [10.35]	66.12 [10.24]	66.43 [10.18]	64.32 [10.34]	63.80 [8.92]	64.00 [8.64]	63.09 [9.84]	64.07 [9.04]	64.25 [8.78]	63.49 [9.82]
*p*-value			.092^a^			<.001^a^			.108^a^			.101^a^
PHQ-9 [SD]^e^	4.4 [3.87]	4.3 [3.73]	4.9 [4.84]	3.9 [3.57]	3.8 [3.47]	4.5 [4.07]	3.4 [3.4]	3.5 [3.5]	3.3 [3.01]	3.4 [3.35]	3.4 [3.44]	3.4 [3.02]
*p*-value			.211^a^			<.001^a^			.326^a^			.876^a^

*Notes*: usHRV, HRV derived from 10 s ECG; SHIP-TREND-0, HRV derived from polysomnography before falling asleep [5 min]; SHIP-TREND-1, HRV derived from rest before probing orthostatic reaction [5 min]; WHtR, Waist-to-Height-Ratio; RMSSD, Root mean square of successive difference between heart beats; HR 5 min, heart rate derived from 5 min ECG recording; HR 10 s, heart rate derived from 10 s ECG; PHQ-9, depression module of the Patient Health Questionnaire; PSG, polysomnography; OT: orthostatic test.

^a^ Student's *t*-test.

^b^ Mann–Whitney *U*-Test.

^c^ SHIP-TREND-0: two missing values in PSG subsample, 11 missing values in usHRV complete sample; SHIP-TREND-1: 1 missing values in OT subsample, 6 missing values in usHRV complete sample.

^d^ SHIP-TREND-0: 2 missing values in usHRV complete sample; SHIP-TREND-1: 1 missing value in usHRV complete sample and 7 missing values in OT subsample.

^e^ SHIP-TREND-0: 12 missing values in PSG subsample subsample, 190 missing values in usHRV complete sample; SHIP-TREND-1: 2 missing values in usHRV complete sample.

#### 2.5.7. Subgroups

To additionally evaluate the validity of usHRV, all samples were divided into a group with and without health issues, resulting into two subgroups (see Table 1 for sample characteristics and demographic data): Thus, while the *complete* samples was comprised of all participants of the corresponding dataset, the subgroup *healthy* consisted of all volunteers who self-reported no mental nor physical health problem. The other subgroup *health issue* consisted of all participants who self-reported any mental disorder, physical health problems (tumors, epilepsy, cold or pain during examination, polyneuropathy, chronic pain, multiple sclerosis, epilepsy, Parkinson's disease, neurological disorders, diabetes,) that might affect HRV or its estimation.

### 2.6. Statistical analyses

All statistical analyses were performed using R 4.2.1 (62). For all regression analysis, the HRV, respectively usHRV, parameter RMSSD was transformed using the natural logarithm to ensure normal distribution (lnRMSSD). Moreover, for modeling non-linear associations, restricted cubic splines, implemented in the *rms* package (63), were used and effects of single variables were assessed by Analyses of variance of type 2. Overall, a significance level of *p* < .05 was used. Sample sizes for linear regressions varied slightly due to missing values (max. 1.27% missing cases).

Differences in sample characteristics between subgroups were tested using either Student's *t*-test or Mann–Whitney *U*-test. The validation of usHRV was performed in several steps:

First, Pearson correlation coefficients were estimated for *usHRV* derived from the 10 s sub-segments of the 5 min ECG with *HRV* obtained from the original 5 min ECG. Besides, correlation coefficients were estimated between *HRV* and matching *usHRV*, derived from distinct ECG recordings, separately for each subsample (PSG subsample, PSG_night subsample and OT subsample) and (sub-)group (complete, healthy and health issues). Moreover, the size of correlations was interpreted according to Cohen's recommendations (*r* < .3 is small, *r* < .5 is medium, *r* ≥ .5 is large) (64). However, strong correlation does not imply close agreement between two measurements as differences in their means are not taken into account. Furthermore, the difference between *HRV* and *usHRV* was modeled as a function of accumulated frequency of the sample.

In a second step, the Bland-Altman procedure was used to calculate the bias and the 95% limits of agreement (LoA) (65, 66). In contrast to standard procedure, HRV estimated from long ECG recording was plotted on the x-axis as a proxy of the gold standard (67).

For further evaluation of usHRV, linear regression analyses were applied in the third step by regressing the HRV parameter *lnRMSSD* (outcomes) on *usHRV*. Additionally, covariates (from the 10 s ECG: heart rate (*HR*), count of valid RR-intervals (*RRcnt*), *daytime* (non-linear); from the long-term ECG: *HR, RRcnt;* time difference between both measurements *(difference in days); WHtR* (non-linear); *age* (non-linear) and *sex* with their interaction *age × sex*) were added in a second model to check whether they might explain this association.

In the fourth step, linear regression analyses were performed separately for each sample (HRV estimated from longer ECG recording matching usHRV, and the complete sample of usHRV) and (sub-)group to investigate the associations between *lnRMSSD* and *sex, age* (non-linear), *age × sex*, *WHtR* (non-linear), as well as *daytime of measurement* (non-linear). Thereby, univariate association of HRV and usHRV, respectively, were calculated with each main effect separately before all variables were introduced together in a multivariate regression model. Then, to evaluate these associations, predicted values for each association of the multivariate analyses were plotted together for visual comparisons. Moreover, the difference in total variance explained by HRV from longer recordings and matching usHRV were calculated by using the toolbox *cocor* (68), which statistically test whether the correlations between actual and predicted values between two measurements are significant different.

In the last step, the association between the *PHQ-9* sum score (outcome) and *lnRMSSD* (non-linear) was evaluated by using linear regression models and a set of covariates (*WHtR* (non-linear), *HR* (non-linear), *daytime of measurement* (non-linear), *age* (non-linear), *sex* and *age × sex*), separately for each HRV-sample and (sub-)group. Univariate associations of *PHQ-9* sum with *HRV* and *usHRV*, respectively, were estimated before covariates were added in a multivariate regression model. Moreover, predicted values of the multivariate analyses were plotted for visual comparison and the explained variance between HRV from longer ECG recordings and matching usHRV was again statistically tested whether they significantly differ from each other, by using the toolbox *cocor*. Furthermore, linear hierarchic regression analysis was performed by comparing each linear model for analyzing matching usHRV (described above) with a linear model that additionally contained *HRV*, *HR* and *daytime of measurement* from the corresponding longer ECG recording to evaluate whether this model explain significantly more variance.

All reported regression coefficients in this manuscript are standardized. Additionally, all analyses in the PSG subsample were repeated with HRV-PSG, derived from the entire PSG recording, to replicate and validate the analysis based on HRV estimated from the corresponding 5 min ECG segment.

## 3. Results

### 3.1. Characteristics of the samples

The characteristics of the samples and their (sub-)groups are presented in Table 1 and Supplementary Table S1. In general, participants with health issues, in contrast to healthy ones, were older and characterized by more males, while WHtR, usHRV and HRV values trended to be higher. Moreover, HR and PHQ-9 sum scores did not differ between these subgroups, apart from slower HR for those with health issues in the OT subsample of SHIP-TREND-1 and more reported depressive symptoms in the complete usHRV sample of SHIP-TREND-1.

Please find the analysis of HRV-PSG deviated from the PSG_night subsample in the supplement (Supplementary Tables S1–S7) as well as the accumulated frequency of participants in relation to the difference between usHRV and HRV (Supplementary Figures S1, S2).

### 3.2. Internal validation

#### 3.2.1. Person correlation

Overall, usHRV, derived from 10 s ECG, was highly correlated with HRV measures derived from longer ECG recordings (see Table 2, Supplementary Tables S2 and S3). Segmenting the original 5 min ECGs in 30 10 s segments and correlating the obtained usHRV with the original HRV showed a mean correlation of *r* = .71 (range: .64–.75) for the PSG subsample and a mean correlation of *r* = .73 (range: .57–.79) for the OT subsample (see Supplementary Table S2 for more details).

**Table 2:** Correlation and bias between HRV and usHRV.

Wave		SHIP-TREND-0			SHIP-TREND-1		
Sample		Polysomnography subsample [5 min]			Orthostatic test subsample [5 min]		
Subgroup		Complete	Healthy	Health issues	Complete	Healthy	Health issues
*N*		1,041	930	111	1,676	1,317	359
Difference in days	Median	8	8	11	27	27	26
	Range	0–1214	0–1214	0–805	0–860	0–860	0–808
Recording start time HRV	Median	22:32	22:32	22:29	10:20	10:20	10:23
	Range	19:57–1:23	20:42–1:23	19:57–00:17	6:37–17:15	6:40–17:15	6:37–16:59
Recording start time - usHRV	Median	12:07	12:02	12:33	10:25	10:23	10:27
	Range	8:35–17:30	8:35–17:30	8:57–15:35	7:47–15:55	7:47–15:45	7:49–15:55
Correlation with usHRV	*r*	.597	.561	.700	.616	.531	.694
	95% CI	.56–.63	.54–.60	.58–.78	.59–.64	.49–.57	.64–.74
	*p*	1.4e-101	4.4e-78	2.1e-17	1.8e-175	9.5e-97	5.9e-53
Bland-Altman	Bias	0.268	0.267	0.275	0.102	0.088	0.151
	95%-LoA	−0.93 to 1.47	−0.89 to 1.43	−1.21 to 1.76	−1.09 to 1.30	−1.04 to 1.21	−1.26 to 1.57

*Notes*: usHRV, HRV derived from 10 s ECG; SHIP-TREND-0, HRV derived from polysomnography before falling asleep [5 min]; SHIP-TREND-1, HRV derived from rest before probing orthostatic reaction [5 min]; CI, confidence interval; LoA, limits of agreement.

Comparing usHRV and HRV derived from distinct ECGs (see Table 2 and Supplementary Table S3), revealed a high correlation of *r* = .60 in the complete PSG subsample in SHIP-TREND-0, while the correlation was slightly smaller for the healthy subgroup (*r* = .56), and larger for those with health issues (*r* = .70). As HRV and PSG-HRV were highly correlated with each other in SHIP-TREND-0 (complete sample: *r* = .78, *p* = 1.2e-209; healthy: *r* = .77; *p* = 3.8e-182; health issues: *r* = .81, *p* = 2.8e-27), similar correlations were found between PSG-HRV and usHRV (*r* = .57; healthy: *r* = .52; health issues: *r* = .75; see Supplementary Table S3 for more details). Also for the OT subsample in SHIP-TREND-1, usHRV correlated highly with HRV (*r* = .62), while the correlation for the healthy subgroup was again slightly smaller (*r* = .53) and slightly higher in the health issue subgroup (*r* = .69).

#### 3.2.2. Bland–Altman plots

**Figure 2:**
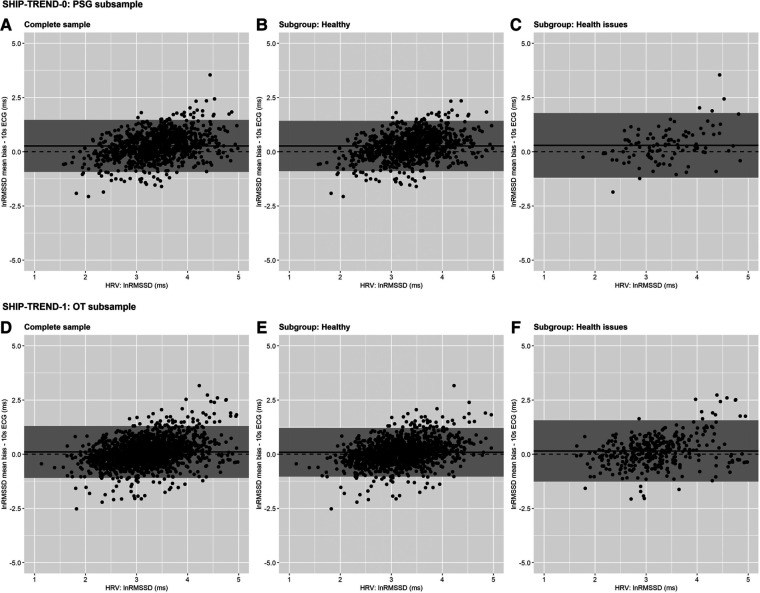
Bland–Altman plots—Measurement error for the log-transformed values of RMSSD calculated as the difference between HRV based on long-term ECG and matching usHRV based on 10 s ECG, separately for different study waves and subgroups. SHIP-TREND-0 PSG subsample: (**A**) complete, (**B**) healthy, (**C**) health issues; SHIP-TREND-1 OT subsample: (**D**) complete, (**E**) healthy, (**F**) health issues. The log-transformed RMSSD derived from 5 min ECG (SHIP-TREND-0: HRV derived from polysomnography before falling asleep; SHIP-TREND-1: HRV derived from rest before probing orthostatic reaction). HRV is plotted on the x-axis and the bias of the usHRV on the y-axis. The grey shaded area indicates the 95% limits of agreement, the dashed horizontal line represents the reference of no bias (*y* = 0), and the mean bias is shown by the continuous horizontal line. *Notes*: usHRV, HRV derived from 10 s ECG; SHIP-TREND-0, HRV derived from Polysomnography before falling asleep [5 min]; SHIP-TREND-1, HRV derived from rest before probing orthostatic reaction [5 min]; lnRMSSD, natural logarithm of root mean square successive difference (RMSSD) between heart beats; PSG subsample, sample consisting of participants who attended the polysomnography in SHIP-TREND-0; OT subsample, sample consisting of participants who attended the orthostatic reaction test in SHIP-TREND-1.

Overall, the bias between HRV and usHRV was higher in the PSG subsample (complete sample: 0.27, Figure 2A; healthy: 0.27, Figure 2B; health issues: 0.28, Figure 2C) than in the OT subsample (complete sample: 0.10, Figure 2D; healthy: 0.09, Figure 2E; health issues: 0.15, Figure 2F), while most usHRV values were within the 95%-LoA (see Supplementary Table S3).

#### 3.2.3. Regression analyses associating usHRV and HRV

Overall, usHRV was positively associated with HRV in the SHIP-TREND-0 and SHIP-TREND-1 subsamples (see Supplementary Table S4; PSG subsample: *β* = .56; OT subsample: *β* = .59), whereas the associations were similar in the healthy subgroups (PSG subsample: *β* = .56; OT subsample: *β* = .57) and for participants with health issues (PSG subsample: *β* = .55; OT subsample: *β* = .63). Taking covariates into account, *β*-values were overall smaller (PSG subsample: *β* = .42; OT subsample: *β* = .50). Interestingly, they were slightly smaller in the healthy subgroups (PSG subsample: *β* = .40; OT subsample: *β* = .42) and higher in the health issues subgroups (PSG subsample: *β* = .50; OT subsample: *β* = .56).

### 3.3. External validation

#### 3.3.1. Regression analyses of usHRV and HRV

Evaluating the association between HRV and age, sex, age × sex, daytime and WHtR across usHRV and HRV separately for each (sub-)group, revealed mostly similar results in particular for age, sex and WHtR, along with no significant difference in the amount of explained variance (associations of age, sex and WHtR based on multivariate models are described below, univariate associations and statistics of all covariates of multivariate analyses are reported in Supplementary Tables S5, S6, see Supplementary Figures S4–S9 for visualization of all models in the Supplementary Material).

**Figure 3:**
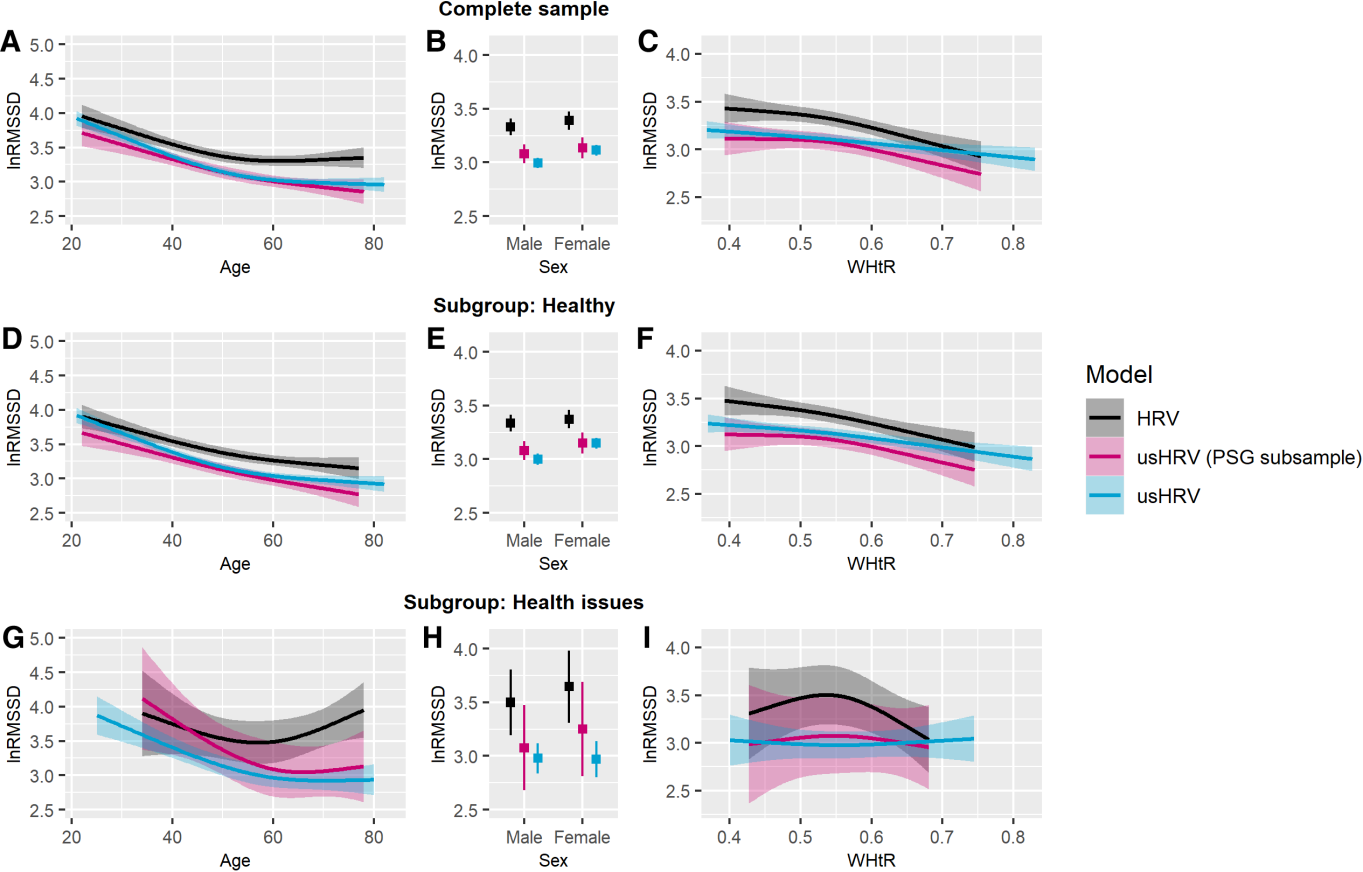
Predicted lnRMSSD as a function of age, sex and WHtR adjusted for daytime. RMSSD was log-transformed, and the plotted values were predicted after adjustment for covariates. Complete sample—association of lnRMSSD with (**A**) age, (**B**) sex, and (**C**) WHtR. Healthy subgroup—association of lnRMSSD with (**D**) age, (**E**) sex, and (**F**) WHtR. Subgroup with health issues—association of lnRMSSD with (**G**) age, (**H**) sex, and (**I**) WHtR. Transparent colored areas are indicating the 95% confidence interval. *Notes*: HRV, HRV derived from polysomnography before falling asleep [5 min]; usHRV, ultrashort HRV estimated from 10 s ECG; usHRV (PSG subsample), usHRV matching the polysomnography subsample.

**Figure 4:**
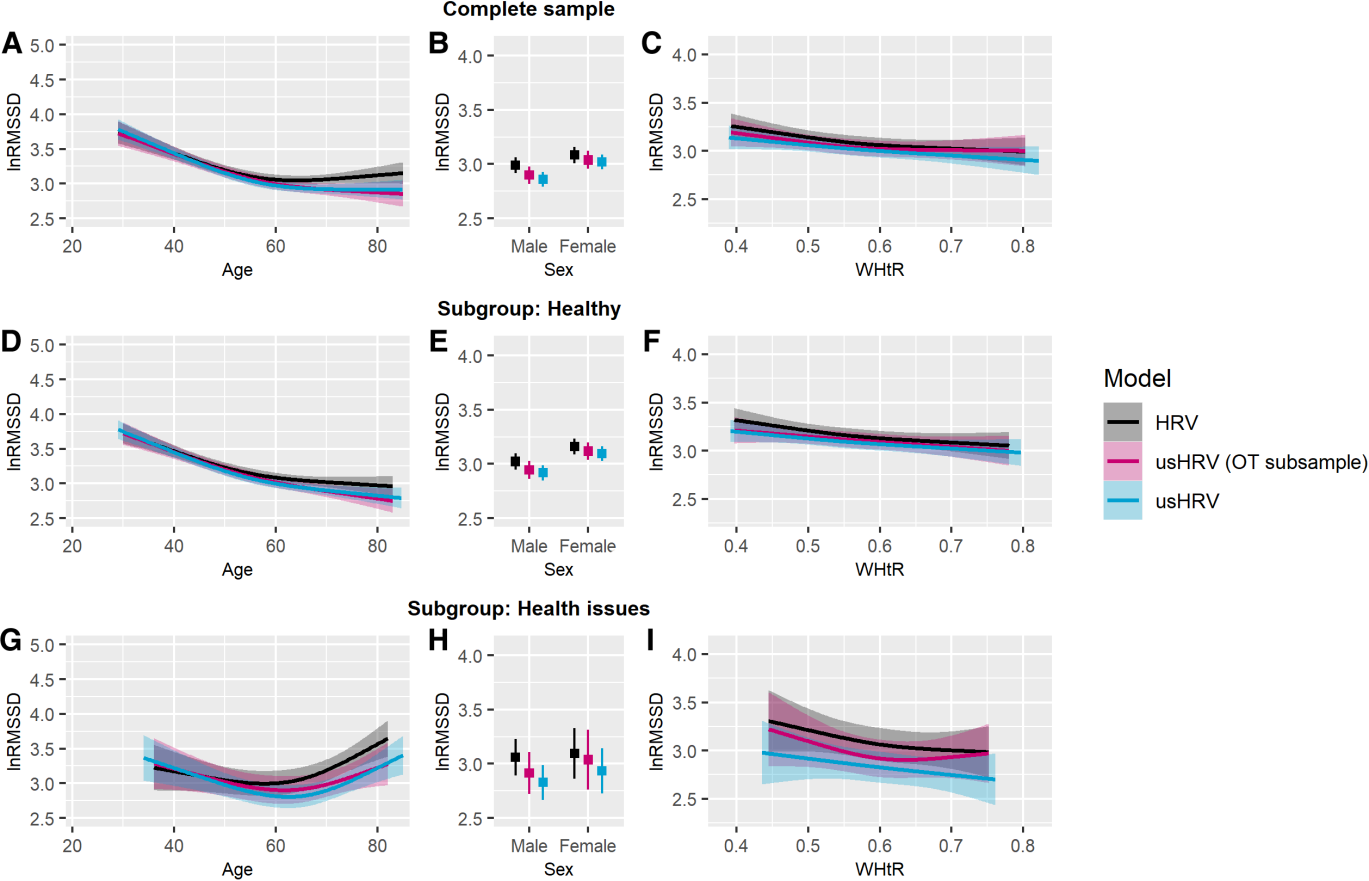
Predicted lnRMSSD as a function of age, sex and WHtR adjusted for daytime. RMSSD was log-transformed, and the plotted values were predicted after adjustment for covariates. Complete sample—association of lnRMSSD with (**A**) age, (**B**) sex, and (**C**) WHtR. Healthy subgroup—association of lnRMSSD with (**D**) age, (**E**) sex, and (**F**) WHtR. Subgroup with health issues—association of lnRMSSD with (**G**) age, (**H**) sex, and (**I**) WHtR. Transparent colored areas are indicating the 95-% confidence interval. *Notes*: HRV, HRV derived from rest before probing orthostatic reaction [5 min]; usHRV, ultrashort HRV estimated from 10 s ECG; usHRV (OT subsample), usHRV matching the orthostatic test subsample.

Overall, a decrease in HRV was observed with increasing age in all samples and the corresponding healthy subgroups (SHIP-TREND-0: see Figures 3A,D; SHIP-TREND-1: Figures 4A,D). The pattern of decreasing HRV was similar for HRV and matching usHRV in the PSG subsample and the OT subsample, albeit the RMSSD-values were generally higher for HRV in SHIP-TREND-0. However, a slight increase or stagnation in HRV was observed for participants older than 60 years in all complete samples, which was mainly driven by the corresponding health issue subgroup (SHIP-TREND-0: see Figure 3G; SHIP-TREND-1: see Figure 4G).

For SHIP-TREND-0, no sex difference was observed for HRV nor matching usHRV in the PSG subsample and neither in the subgroups (see Figures 3B,E,H). In contrast, women had higher values of usHRV in the complete sample of usHRV, which was driven by the healthy subgroup. Moreover, women in SHIP-TREND-1 were characterized by higher HRV and usHRV values in all complete samples and healthy subgroups (see Figures 4B,E).

Besides, using univariate analyses, WHtR was found to be associated with HRV and usHRV in all complete samples and healthy subgroups. Using multivariate analyses, covariates altered these associations. Thus, HRV was found to be lower with higher WHtR in the PSG subsample of SHIP-TREND-0 and in the healthy subgroup, which was similar for matching usHRV (see Figures 3C,F). This association pattern was also observed in the complete sample of usHRV and in its healthy subgroup. In SHIP-TREND-1, lower HRV was also negatively associated with higher WHtR in the complete OT subsample and the healthy subgroup (see Figures 4C,F). Moreover, a similar association trended for matching usHRV (complete sample: *p* = .062; healthy subgroup: *p* = .051) and was similar in the complete sample and healthy subgroup of usHRV. Besides, there was no association between HRV nor usHRV for participants with health issues in SHIP-TREND-0 and SHIP-TREND-1 (see Figures 3I, 4I).

The amount of explained variance was not significant different between HRV and matching usHRV, neither in the PSG subsample of SHIP-TREND-0 nor in the OT subsample of SHIP-TREND-1.

#### 3.3.2. PHQ-9

**Figure 5:**
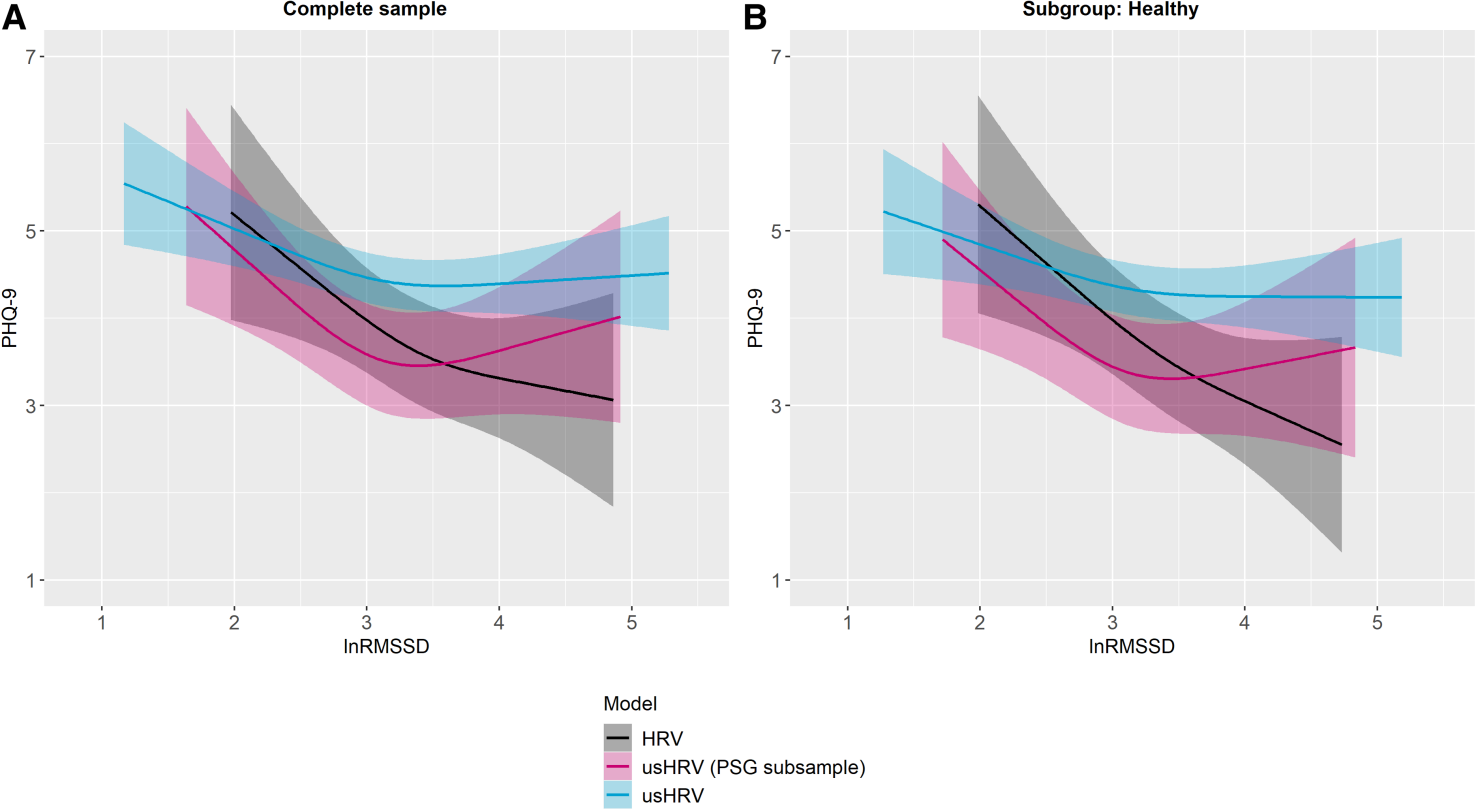
Predicted PHQ-9 sum score by lnRMSSD, adjusted for age, sex, HR, WHtR and daytime. Plotted values were predicted after adjustment for covariates. Associations of the PHQ-9 sum score and lnRMSSD, separately for HRV, usHRV (PSG subsample) and usHRV: (**A**) complete sample, and (**B**) healthy subgroup. Transparent colored areas are indicating the 95% confidence interval. *Notes*: HRV, HRV derived from polysomnography before falling asleep [5 min]; usHRV, RMSSD derived from 10 s ECG; PHQ-9, depression module of the Patient Health Questionnaire; usHRV (PSG subsample), usHRV matching the polysomnography subsample.

Univariate regression analyses showed only a significant association of the depression score PHQ-9 with HRV in the subgroup of health issues in SHIP-TREND-1 (see Supplementary Table S5). In contrast, multivariate regression analyses revealed for SHIP-TREND-0 that PHQ-9 was significantly associated with HRV and usHRV, respectively, in the complete samples and the healthy subgroups. These findings were similar and showed no significant difference in the amount of explained variance (see Supplementary Table S7 for statistics, see Figure 5 for visualization of significant associations and see Supplementary Figures S10–S1S5 for visualization of all associations).

HRV was found to be lower in individuals with higher PHQ-9 scores in the PSG subsample and the corresponding healthy subgroup in SHIP-TREND-0. This association pattern was similar for matching usHRV (see Figures 5A,B) as it was similar for usHRV values smaller than 3.5, while higher values stagnated or even showed a slightly positive association pattern. A similar association was observed in the complete usHRV sample and its healthy subgroup in SHIP-TREND-0. Besides, no association was found in the health issue subgroup, similar for HRV and usHRV (see Supplementary Figures S10M). In contrast to SHIP-TREND-0, no association of HRV with scores on the PHQ-9 was found in the OT subsample nor in the corresponding healthy subgroup in SHIP-TREND-1 (see Supplementary Figures S14A,G). In line, no association was found between usHRV and PHQ-9 scores, neither for matching usHRV or the complete usHRV sample. However, more reported depressive symptoms were associated with lower HRV in the health issue subgroup in SHIP-TREND-1 (see Supplementary Figure S14M), but not for the matching usHRV nor for the complete usHRV sample.

Moreover, testing whether linear regression models associating PHQ-9 scores with usHRV would explain significantly more variance by adding corresponding HRV derived from 5 min ECGs, including covariates, revealed no significant improvement, neither in the PSG subsample or OT subsample, except for the health issue subgroup of the latter subgroup.

Above all, no significant difference in the amount of explained variance between any HRV and matching usHRV was found, neither in the PSG subsample nor in the OT subsample.

## 4. Discussion

The present study investigated the applicability and validity of the HRV parameter RMSSD based on 10 s multichannel ECG recording (usHRV) as a proxy for vagal-mediated HRV in a large epidemiological cohort. Our results revealed high correlations between usHRV and HRV based on multiple long-term ECGs, whereby usHRV was the strongest predictor for HRV in all samples and (sub-)groups. Additionally, similar associations for age, sex and WHtR were found with usHRV and HRV, while no difference in the amount of explained variance was observed. Furthermore, this study demonstrated similar findings for usHRV in association with depressive symptoms, illustrating the possibilities of usHRV for risk prediction.

In detail, correlations between usHRV derived from 10 s sub-segments of 5 min ECG and HRV obtained from the entire 5 min were high but not perfect. Interestingly, these correlations were only slightly higher than the correlations found between separated recorded and derived 10 s usHRV and HRV, probably driven by differences of the ECG recording (e.g., setting). While the correlations between different HRV origins were lower than in most studies that compared usHRV based on a 10 s segment with HRV from the same ECG (36, 37), our results were within the range reported by Nussinovitch (42) and Boos et al. (43), the latter using two separate ECGs for comparison. In this regard, our findings are worth of remark as the ECGs in this study were not directly performed according to the HRV guidelines (28, 29)—it was only assured that the participants were at rest. Most importantly, usHRV remained a strong predictor for HRV across all subsamples and (sub-)groups while additionally taking covariates into account, like HR from the long-term ECGs which is *per se* associated with HRV (69, 70). Moreover, as compared ECGs were recorded several days up to years apart and as these correlations were only slightly smaller than those between sub-segmented 10 s usHRV and the original 5 min HRV, our findings are in line with the assumption that vagal-mediated HRV is stable over time (13, 14) as well as that HRV can be estimated from 10 s multichannel ECGs that are typical performed in clinical settings and epidemiological studies.

This conclusion is further supported by our findings of similar association patterns of HRV and usHRV with age, sex and WHtR, as well as no difference in the amount of explained variance by HRV and usHRV, further supporting the validity of usHRV. Specifically, HRV and usHRV were found to be similar lower in older people in all samples and healthy subgroups, while this association tended to flatten in an exponential manner with increasing age, possibly driven by a selection and survival bias typically seen in population-based studies (71). Besides, this is in line with previous studies, reporting a negative association between HRV and age for HRV based on 5 min ECG (48), long-term HRV (49) and usHRV (72). Moreover, the observed decline of autonomic regulation with increasing age has been discussed to promote a variety of age-related cardio-vascular conditions and mortality (73), as well as, to might go along with an accelerated decline of cognitive performance that could increase the risk for Alzheimer or other dementias (74–77). However, and surprisingly, individuals with health issues older than approximately 55–60 years showed increasing HRV and usHRV-values. This is somehow contra-intuitive and in contrast to the findings for the corresponding healthy subgroups and therefore needs further investigation to clarify its origin. It might be caused by a selection bias (71), medication, which is more likely to be taken by older and sick people (6, 78, 79), or undiagnosed CVDs that may have affected the spontaneous sinus rhythm of the heart.

Furthermore, mostly similar results regarding sex differences were found between HRV and usHRV, however, they differed between study waves, samples and (sub-)groups. Interestingly, previous research reported also heterogenous findings for the HRV parameter RMSSD regarding sex differences (80). In contrast, studies using frequency-based methods, reported more consistently higher vagal tone for women indexed by high frequency power of HRV (80), which might contribute to a later onset of CVD and CVD-related mortality (81).

Besides, the association patterns of higher WHtR with lower HRV were mostly similar between HRV derived from long-term ECG and usHRV in all samples, except for usHRV in the OT subsample. These results are replicating previous findings, demonstrating negative association between RMSSD and WHtR (47, 50), indicating ANS dysfunction in people with abnormal body weight. For example, a variety of studies reported a parasympathetic dominance in individuals with anorexia nervosa (82, 83), while individuals with obesity were characterized by a more reduced vagal tone (84).

Additionally, our findings demonstrated similar association patterns of depressive symptoms and HRV in both study waves with no differences in the amount of explained variance. In SHIP-TREND-0, higher HRV values were associated with less symptoms of depression in the PSG subsample and the complete sample of usHRV as well as their healthy subgroups. In contrast to our assumptions, no association at all was found in SHIP-TREND-1, presumably partly due to a selection bias in this cohort and/or higher mean age that is associated with a decrease in depressive symptoms. Thus, a decreased ANS flexibility together with a higher number of healthy individuals could disguise this association. Nevertheless, our findings demonstrate similar finding for usHRV and HRV derived from long-term ECGs regarding depressive symptoms, which is further supported as no relevant differences in fraction of explained variance were found and HRV from longer ECGs did not explain additional variance above usHRV. The observed association of more depressive symptoms with lower HRV in SHIP-TREND-0 is in accordance with previous studies, reporting vagal-mediated HRV to be lower in individuals with depression in contrast to healthy controls (85–87). Moreover, as already mild depressive symptoms are accompanied by a slight ANS imbalance, this might increase the risk for the emergence of major depression, possibly mediated by negatively affected emotion regulation (88) or inappropriate and inflexible stress responses due to altered tonic vagal influence on the sympathetic (89). Moreover, previous studies demonstrated an association of depression and ANS dysfunction with CVD and mortality (3, 4, 15). Therefore, it would be important to investigate to which degree already mild non-pathological depressive symptoms might increase the risk for depression as well as for physical and other mental health problems to develop effective prevention programs.

Besides, we performed an additional analysis with HRV derived from overnight ECG (PSG-HRV) to validate our results in the PSG subsample. Most importantly, PSG-HRV and HRV were highly associated in the PSG_night subsample and the association between HRV and usHRV were replicated. Moreover, association patterns with age, sex and depressive symptoms were mostly similar, however, not for WHtR. These deviating results could potentially be driven by different mechanisms and effects during sleep (e.g., sleep quality and architecture, obstructive sleep apnea) (90, 91).

### 4.1. Limitation

Our study has some limitations. We only used ECGs that were recorded at rest/sleep in clinical settings as an approximation to the gold standard defined by current guidelines. Therefore, we might not be able to fully assess the actual amount of vagal control, resulting into a potential bias and less power for our analysis. Therefore, it would be important to replicate our results with an HRV measurement in accordance with the HRV guidelines. However, as the association between usHRV and HRV was high across all samples and we were able to replicate previous findings, we assume that our used ECGs were close to the gold standard.

Besides, very low and very high usHRV values should be handled with caution, as they might be slightly biased as changes in a single RR-interval contributes a significant amount of information regarding the obtained usHRV.

Moreover, we only used the HRV parameter RMSSD as previous studies recommended longer recording periods for other HRV parameters (36, 40), as spectral analysis is seeming to perform not well in heart series of less than 20 beats (92). However, there is some evidence that HF-HRV could already be estimated from 20 s ECG (33) and might be valid from 10 s ECGs depending on age (36). Thus, further research on evaluating the applicability and validity of different HRV parameters estimated from 10 s ECG in epidemiological studies is needed.

As approximately only one third of the SHIP-TREND-0 population underwent PSG and only approximately two third of the SHIP-TREND-1 population participated orthostatic testing, the association analyses between HRV and usHRV might be affected by a selection bias, as for example Fietze et al. (90) found that the PSG data set was slightly healthier and more educated. Although our analyses accounted for different covariates, our requested data set was limited and thus we were not able to consider additional covariates which might be associated with HRV.

Besides, we did not take medication, its changes between the ECG recording sessions, or other health impairments (e.g., cardiometabolic diseases) into account that might have affected HRV, especially in older participants. Moreover, our health issue subgroups were relatively small and were characterized by a heterogeneity of health problems. Because of that, our findings for these subgroups are limited and should be interpreted with caution.

Since it is not possible to test whether the linear regression models for usHRV and HRV are statistically equal, only the difference between models could be tested. Therefore, the visual comparison should be interpreted with caution. Moreover, as the results especially for WHtR and PHQ-9 showed alterations between univariate and multivariate analyses, further analyses are needed to explore this (e.g., using mediation or moderation analyses).

## 5. Summary and conclusion

In this paper we have replicated and extended previous findings between vagal-mediated HRV and demographic variables as well as depressive symptoms by using ECGs that were performed in epidemiological settings. Moreover, for the first time we have systematically evaluated these results with usHRV derived from 10 s multichannel ECG in a large population-based cohort using a newly developed HRV-pipeline. Thereby, we presented information about the validity and applicability of usHRV as a proxy for vagal-mediated HRV, which can be derived from 10 s ECGs that are typical performed in clinical and epidemiological settings and are sufficiently reliable at the same time. Our results closing a big lack of knowledge by evaluating and providing arguments for the usage of 10 s usHRV in epidemiological studies where longer ECGs are rarely applied. Moreover, this allows to address new research questions, for example in a longitudinal manner, even when only 10 s ECGs are available. Thus, using 10 s usHRV could help to investigate potential risk factors that might contribute to the emergence of future mental and physical health problems in more detail in population-based cohorts, like for example the German National Cohort (NAKO) (93) or UK-Biobank (94). Therefore, estimated HRV values from this study will be available as endpoint data in the SHIP cohorts for further analyses, for example, risk predicting of CVD events.

Because of the higher susceptibility of 10 s usHRV for biases during recording, further investigation, and replication, especially using one HRV measurement according the HRV guidelines as a gold standard, is needed. Despite this, at this moment we recommend the usage of 10 s usHRV in large samples and only with a subsample of HRV based on a long-term ECG recording for validation to prevent false positive or negative findings. Nevertheless, longer ECGs of at least 20–30 s would be preferable for reasons of validity and the possibility to estimate different HRV parameters.

## Data Availability

The datasets presented in this article are not readily available because the authors do not have the permission to share the data. Requests to access the datasets should be directed to Transfer Unit for Data and Biomaterials of the University Medicine Greifswald (https://transfer.ship-med.uni-greifswald.de/FAIRequest/).
