# Supplementary material for: Evaluating heart rate variability with 10 second multichannel electrocardiograms in a large population-based sample
Source: Front Cardiovasc Med. 2023 May 12;10:1144191. doi: 10.3389/fcvm.2023.1144191 (PMC10213655; doi:10.3389/fcvm.2023.1144191)
Supplement: Supplementary file 1 [file Presentation1.pdf]

Supplementary Material

Evaluating heart rate variability with 10 second multichannel electrocardiograms in a large population-based sample

Elischa Krause*, Marcus Vollmer, Katharina Wittfeld, Antoine Weihs, Stefan Frenzel, Marcus Dörr, Lars Kaderali, Stephan B. Felix, Beate Stubbe, Ralf Ewert, Henry Völzke & Hans J. Grabe

*** Correspondence:** Corresponding Author: elischa.krause@uni-greifswald.de

1 Supplementary Data

Supplementary Results

Internal validation

Bland-Altman plots. In contrast to HRV in SHIP-TREND-0, the bias was higher when comparing usHRV and PSG-HRV, albeit again similar for the complete sample (0.37, see Table S3 and Fig. S3D) and both subgroups (healthy: 0.37, Fig. S1E; health issues: 0.35, Fig. S3F).

Regression analyses associating usHRV and PSG-HRV in SHIP-TREND-0. Regression analyses (see Table S4) revealed that usHRV was positively associated with PSG-HRV in the complete sample of the PSG_night subsample of SHIP-TREND-0 (β = .56) and similar in the healthy subgroup (β = .55) and slightly higher in the subgroup health issues (β = .66). Taking covariates into account, β-values were overall smaller (β = .47). Interestingly, they were slightly smaller in the healthy subgroups (β = .43) and higher in the health issues subgroups (β = .69).

Difference between usHRV and HRV and percentage sample. The absolute mean difference of RMSSD values between HRV and matching usHRV in all three subsamples of matching HRV and usHRV was quite similar, although slightly smaller in the SHIP-Trend-1 OT subsample (SHIP-Trend-0: PSG subsample M[SD] = 15.93[16.73]; SHIP-Trend-0: PSG_night subsample M[SD] = 17.22[18.21]; SHIP-Trend-1: OT subsample M[SD] = 13.89[20.09]). Moreover, Figure S1 shows that about 80% of all usHRV values have an absolute difference from HRV of less than 25 ms, which is considered marginal (SHIP-Trend-0: 24; SHIP-Trend-0: 27; SHIP-Trend-1: 19). A similar pattern was observed for the lnRMSSD values: The mean difference between HRV and usHRV in all three subsamples of HRV and matching usHRV was quite similar (SHIP-Trend-0: PSG subsample M[SD] = 0.52[0.42]; SHIP-Trend-0: PSG_night subsample M[SD] = 0.55[0.44]; SHIP-Trend-1: OT subsample M[SD] = 0.46[0.41]). Moreover, Figure S2 indicates that about 80% of all usHRV values have an absolute difference of less than ~0.8 (SHIP-Trend-0: 0.82; SHIP-Trend-0: 0.88; SHIP-Trend-1: 0.72).

External validation

Regression analyses comparing usHRV and PSG-HRV. The association between HRV and age, sex, age x sex, daytime and WHtR across usHRV and PSG-HRV, separately for each (sub-)group, were mostly similar for age, while differences were found for sex and WHtR (see Table S5 and S6).

Overall, a decrease of PSG-HRV with increasing age was observed, which was similar for usHRV and usHRV in the complete sample of the PSG_night subsample and the healthy subgroup (see Fig. S6A and S6E). However, a U-shape association pattern for PSG-HRV was found in the health issue subgroup, showing decreasing PSG-HRV with increasing age for those younger than approximately 55-60 years and increasing PSG-HRV for the older ones. In contrast, usHRV decreased with increasing age in the PSG_night subsample and in

the complete sample of usHRV (see Fig. S6I). Besides, RMSSD-values were generally higher for PSG-HRV than for usHRV.

No sex differences were found in the complete sample for PSG-HRV and matching usHRV (see Fig. S6B). However, women had significant higher usHRV values in the complete sample of usHRV, which was driven by the healthy subgroup (see Fig. S6F). In contrast, younger men had higher PSG-HRV values than women in the healthy subgroup, while this association was reversed for the older participants (see Fig. S7D). This interaction was not seen for matching usHRV (see Fig. S7E).

In contrast to HRV in SHIP-TREND-0, WHtR was not associated with HRV-PSG in the complete sample and the healthy subgroup (see Fig. S6C and S6G). However, higher usHRV values in the sample of matching usHRV and in the complete sample and the healthy subgroup were negatively associated with higher WHtR. Besides, no association at all was found for participants with health issues (see Fig. S6K).

Comparing the amount of explained variance revealed that there were no significant differences between PSG-HRV and usHRV.

PHQ-9. Linear regression analyses associating the depression score PHQ-9 with HRV revealed similar findings between PSG-HRV and usHRV as well as no significant difference in the amount of explained variance (see Fig. S12A, S12G and S12M; Table S5 and S7. Though, higher PSG-HRV was associated trend significant with lower scores on the PHQ-9 in the PSG_night subsample, while this association was significant in the healthy subgroup. Moreover, this association was similar for matching usHRV and the healthy subgroup, although covering a wider range of HRV values. In more detail, a similar pattern of lower PHQ-9 scores and higher HRV was observed for PSG-HRV and matching usHRV values smaller than 3.5, while it seemed to reach a plateau for higher values. Besides, higher usHRV was also associated with lower PHQ-9 scores in the complete usHRV sample and the healthy subgroup, however, the association was not so pronounced.

Testing whether linear regression models associating PHQ-9 scores with usHRV would explain additional variance by adding PSG-HRV, including their covariates, revealed no significant improvement at all. Moreover, no significant difference in the amount of explained variance between PSG-HRV and matching usHRV was found.

2 Supplementary Figures and Tables

2.1 Supplementary Figures

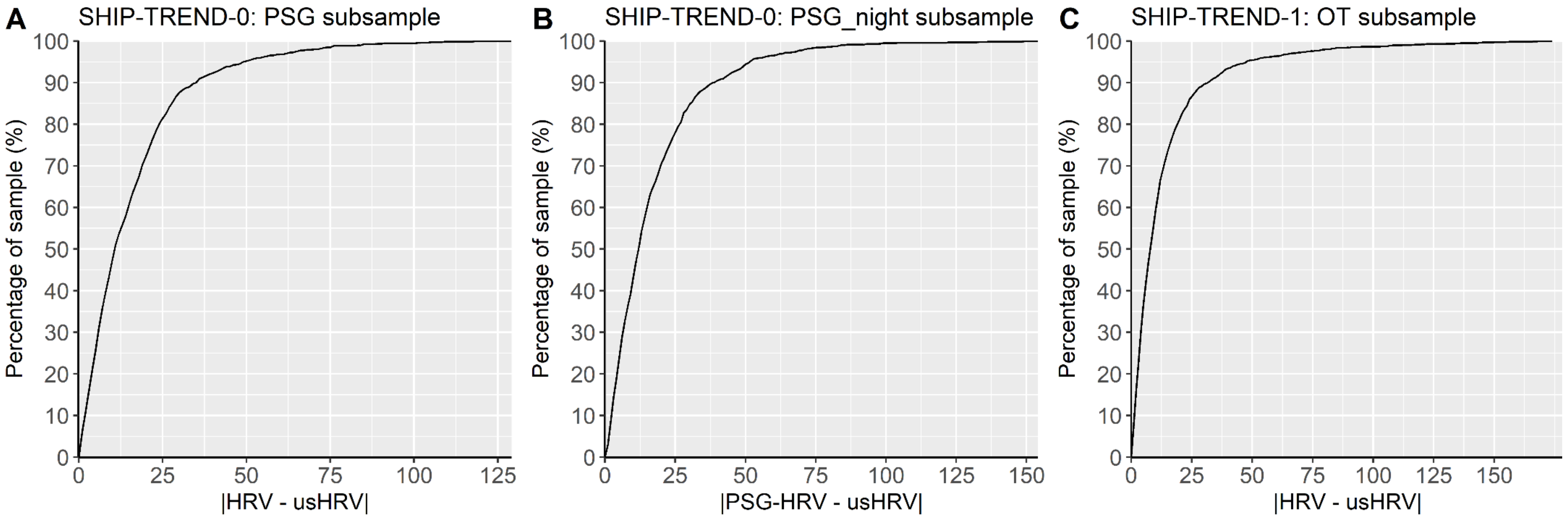

Supplementary Figure S1. Accumulated frequency of participants in relation to the absolute difference between RMSSD values of HRV and matching usHRV. **(A)** HRV derived from polysomnography before falling asleep [5min], **(B)** HRV derived from entire polysomnography [5-11h], **(C)** HRV derived from rest before probing orthostatic reaction [5min].

Notes: usHRV: RMSSD from 10-second ECG; RMSSD: root mean square successive difference between heart beats.

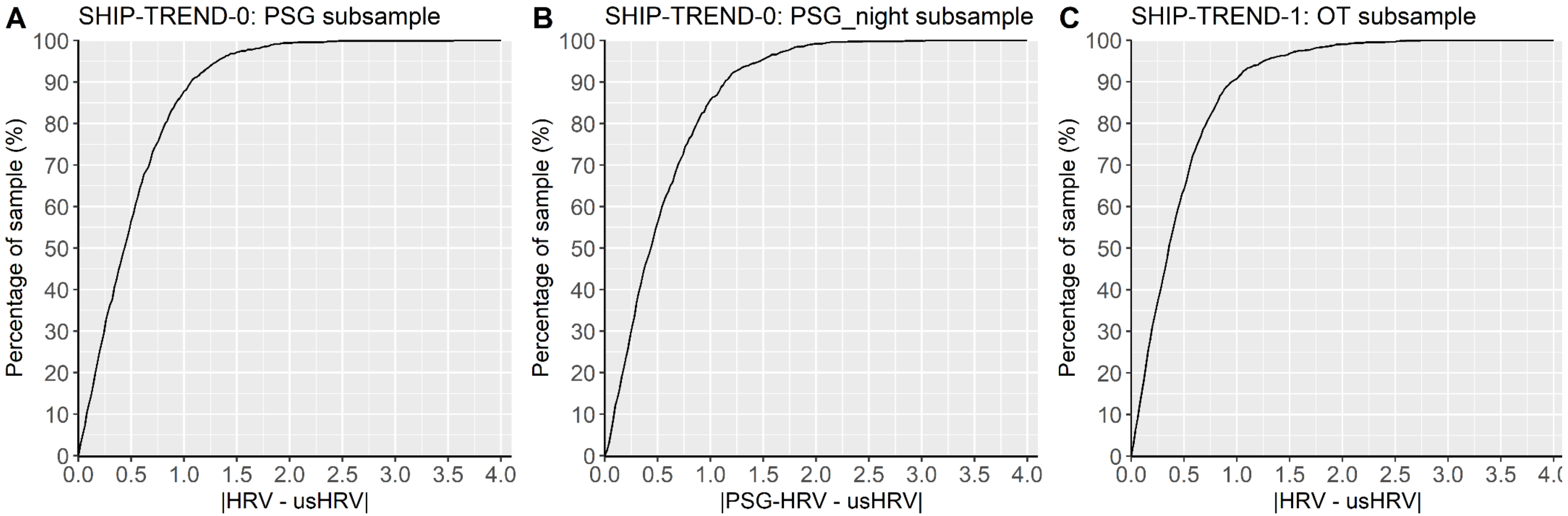

Supplementary Figure S2. Accumulated frequency of participants in relation to the absolute difference between lnRMSSD values of HRV and matching usHRV. **(A)** HRV derived from polysomnography before falling asleep [5min], **(B)** HRV derived from entire polysomnography [5-11h], **(C)** HRV derived from rest before probing orthostatic reaction [5min].

Notes: usHRV: lnRMSSD from 10-second ECG; lnRMSSD: natural logarithm of root mean square successive difference (RMSSD) between heart beats.

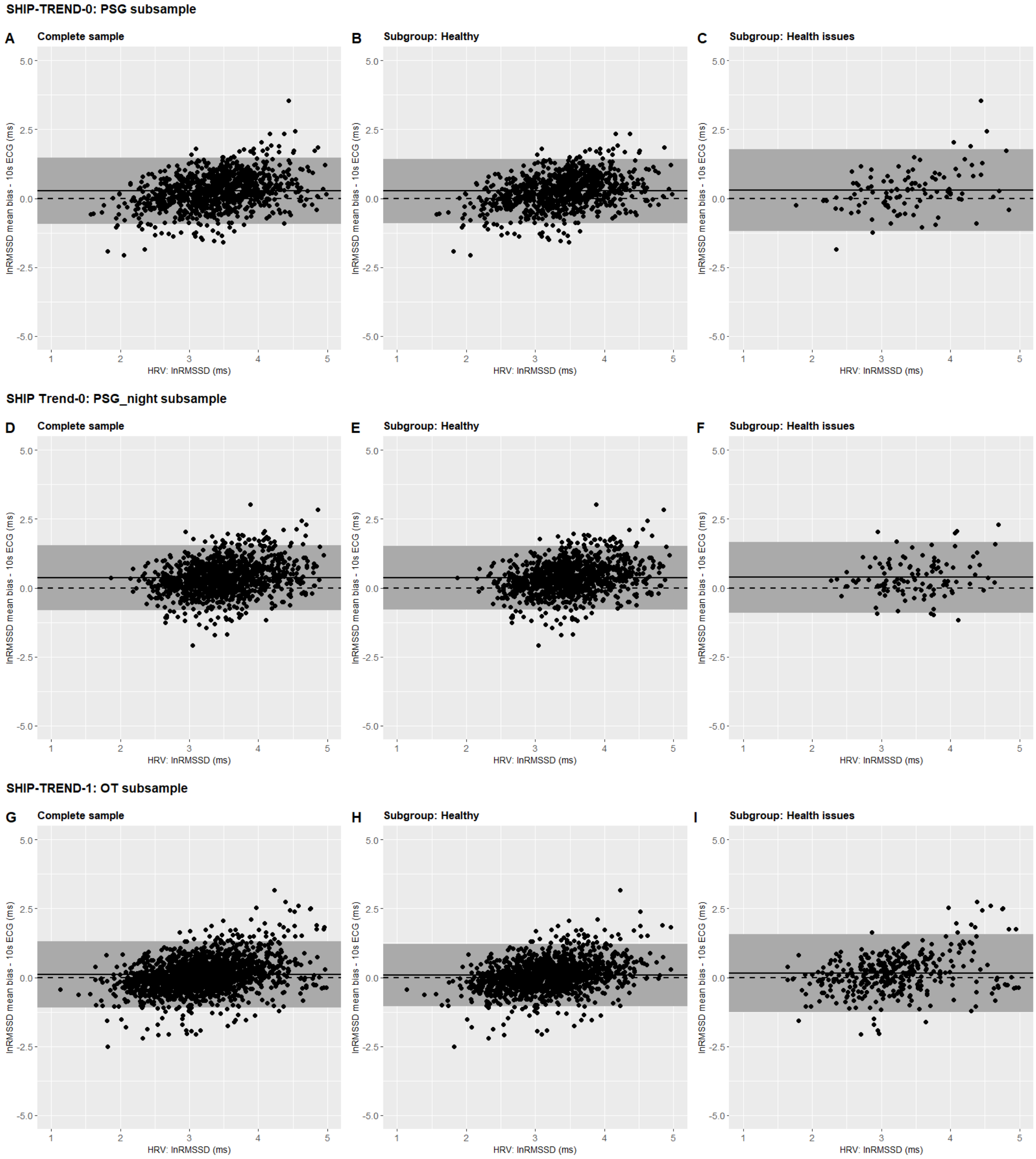

Supplementary Figure S3. Bland-Altman-Plots - Measurement error for the log-transformed values for RMSSD calculated as the difference between HRV based on the long-term ECG and matching usHRV based on 10-second ECG, separately for the different study waves and subgroups. SHIP-TREND-0 PSG subsample [5 min]: **(A)** complete, **(B)** healthy, **(C)** health issues; SHIP-TREND-0 PSG_night subsample [5-11h]: **(D)** complete, **(E)** healthy, **(F)** health issues; SHIP-TREND-1 OT subsample [5min]: **(G)** complete, **(H)** healthy, **(I)** health issues. The log-transformed RMSSD from the longer ECG-recording (SHIP-TREND-0: HRV and PSG-HRV; SHIP-TREND-1: HRV) is plotted on the x-axis and the bias of the usHRV on the y-axis. The grey shaded area indicates the 95% limits of agreement, the dashed horizontal line represents the reference of no bias (y = 0) and the mean bias is shown by the continuous horizontal line.

Notes: SHIP-TREND-0: HRV derived from polysomnography before falling asleep [5min]; PSG-HRV derived from entire polysomnography of the polysomnography_night subsample [5-11h]; SHIP-TREND-1: HRV derived from rest before probing orthostatic reaction [5min]; usHRV: HRV derived from 10-second ECG; lnRMSSD: natural logarithm of root mean square successive difference (RMSSD) between heart beats; PSG subsample: subsample consisting of participants who attended the polysomnography in SHIP-TREND-0 and had valid HRV before falling asleep [5 min]; PSG_night subsample: subsample consisting of participants who attended the polysomnography in SHIP-TREND-0 and had valid HRV for the entire measurement [5-11h]; OT subsample: sample consisting of participants who attended the orthostatic reaction test in SHIP-TREND-1.

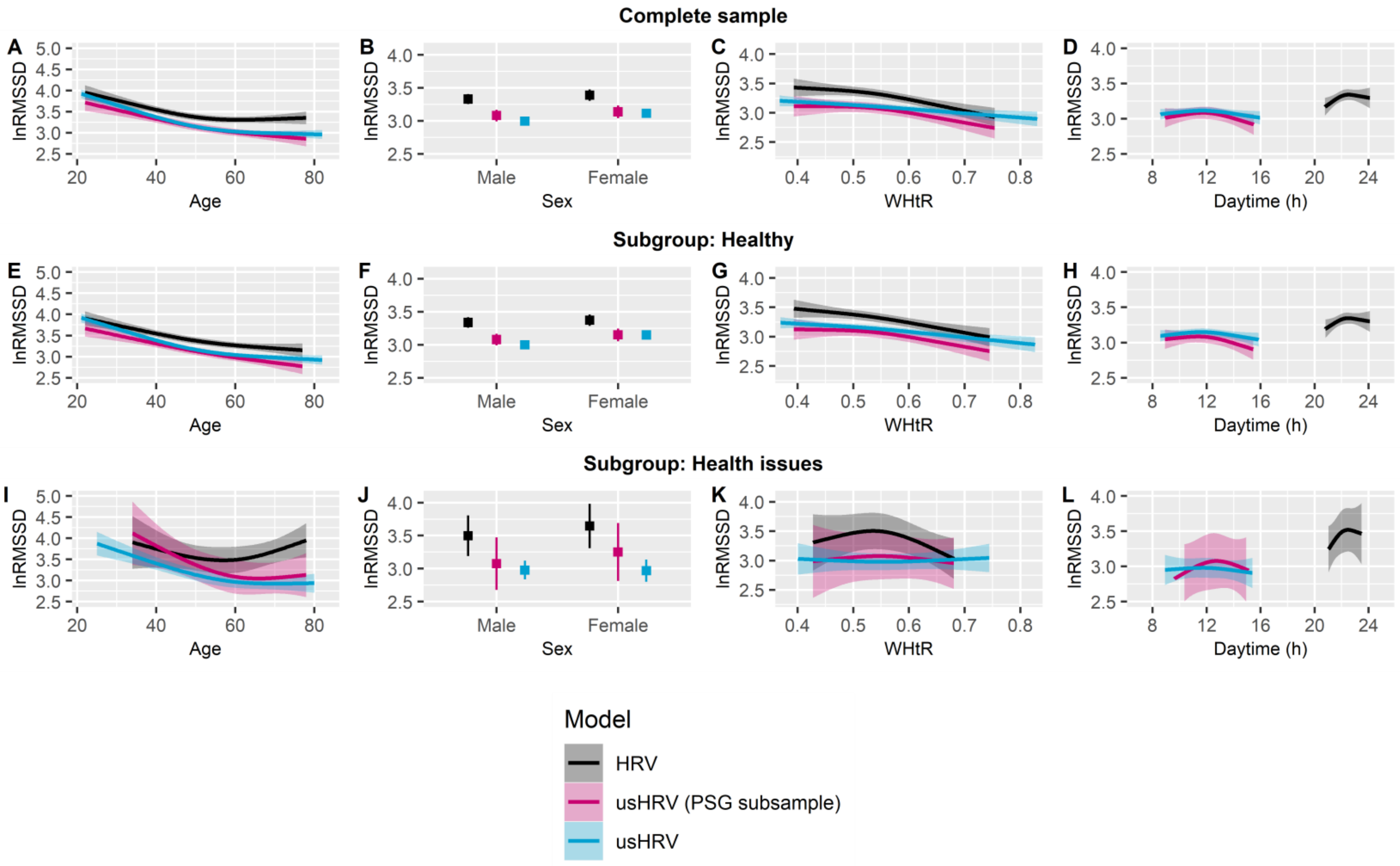

Supplementary Figure S4. Predicted lnRMSSD as a function of Age, Sex and WHtR adjusted for daytime. RMSSD was log-transformed, and the plotted values were predicted after adjustment for covariates. Complete sample – association of lnRMSSD with **(A)** age, **(B)** sex, **(C)** WHtR, and daytime **(D)**. Healthy subgroup – association of lnRMSSD with **(E)** age, **(F)** sex, **(G)** WHtR, and **(H)** daytime. Subgroup with health issues – association of lnRMSSD with **(I)** age, **(J)** sex, **(K)** WHtR, and **(L)** daytime. Transparent colored areas indicating the 95-% confidence interval.

Notes: HRV: HRV derived from polysomnography before falling asleep [5min]; usHRV: ultrashort HRV estimated from 10-second ECG; daytime: Daytime in hours when the ECG-recording was started; lnRMSSD: natural logarithm of root mean square successive difference (RMSSD) between heart beats; usHRV (PSG subsample): usHRV matching the polysomnography subsample [5min].

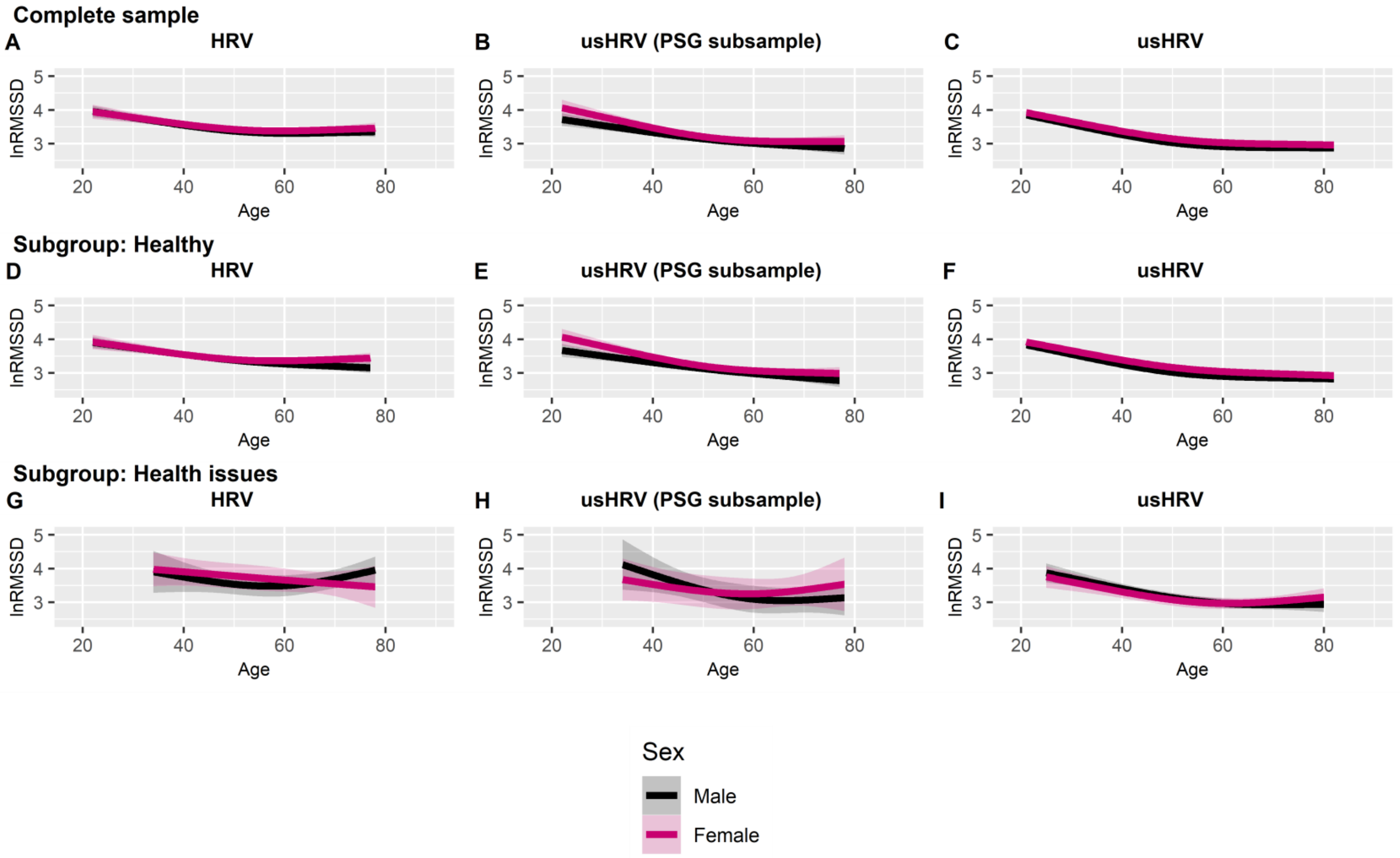

Supplementary Figure S5. Predicted lnRMSSD and the interaction of Age and Sex, adjusted for WHtR and daytime. Plotted values were predicted after adjustment for covariates Complete sample: **(A)** HRV, **(B)** usHRV (PSG subsample), and **(C)** usHRV; Subgroup healthy: **(D)** HRV, **(E)** usHRV (PSG subsample), and **(F)** usHRV; Subgroup health issues: **(G)** HRV, **(H)** usHRV (PSG subsample), and **(I)** usHRV. Transparent colored areas indicating the 95-% confidence interval. *Notes:* HRV: HRV derived from polysomnography before falling asleep [5min]; usHRV: ultrashort HRV estimated from 10-second ECG; daytime: Daytime in hours when the ECG-recording was started; usHRV (PSG subsample): usHRV matching the polysomnography subsample.

Notes: HRV: HRV derived from polysomnography before falling asleep [5min]; usHRV: ultrashort HRV estimated from 10-second ECG; lnRMSSD: natural logarithm of root mean square successive difference (RMSSD) between heart beats.

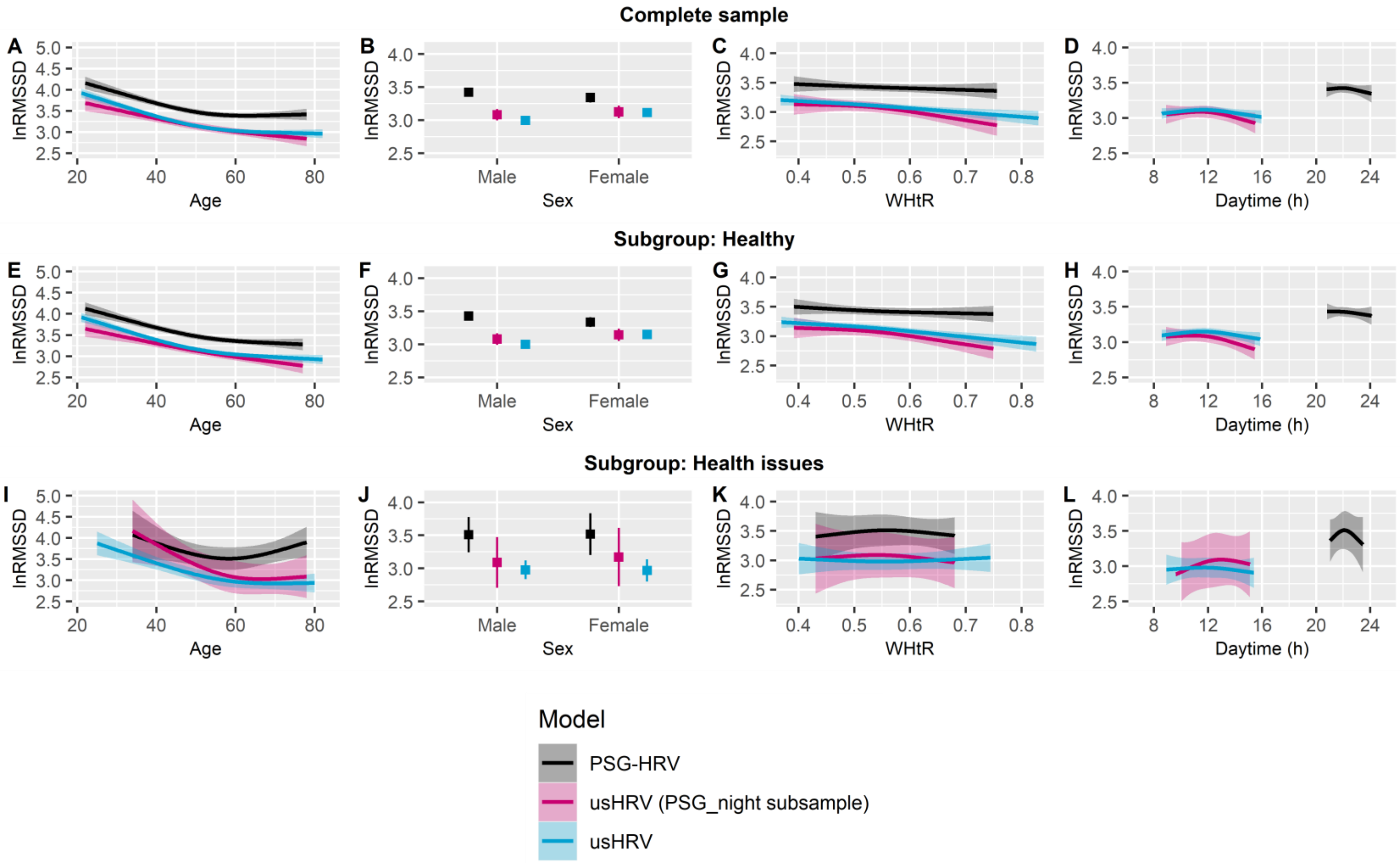

Supplementary Figure S6. Predicted lnRMSSD as a function of Age, Sex and WHtR adjusted for daytime. RMSSD was log-transformed, and the plotted values were predicted after adjustment for covariates. Complete sample – association of lnRMSSD with **(A)** age, **(B)** sex, **(C)** WHtR, and daytime **(D)**. Healthy subgroup – association of lnRMSSD with **(E)** age, **(F)** sex, **(G)** WHtR, and **(H)** daytime. Subgroup with health issues – association of lnRMSSD with **(I)** age, **(J)** sex, **(K)** WHtR, and **(L)** daytime. Transparent colored areas indicating the 95-% confidence interval.

Notes: PSG-HRV: HRV derived from entire polysomnography [5-11h]; usHRV: ultrashort HRV estimated from 10-second ECG; daytime: Daytime in hours when the ECG-recording was started; lnRMSSD: natural logarithm of root mean square successive difference (RMSSD) between heart beats; usHRV (PSG_night subsample): usHRV matching the polysomnography_night subsample.

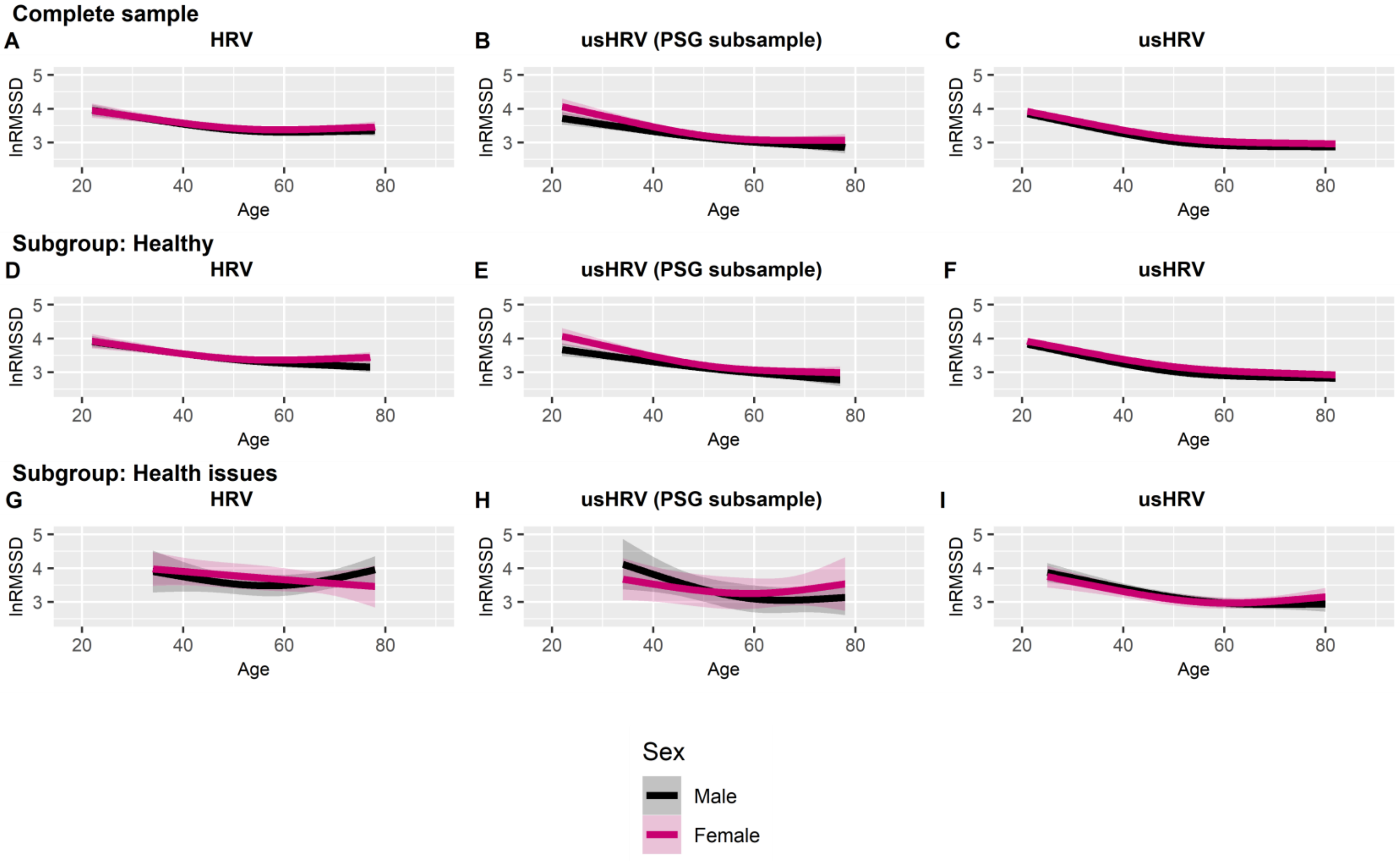

Supplementary Figure S7. Predicted lnRMSSD and the interaction of Age and Sex, adjusted for WHtR and daytime. Plotted values were predicted after adjustment for covariates. Complete sample: **(A)** HRV, **(B)** usHRV (PSG_night subsample), and **(C)** usHRV; Subgroup healthy: **(D)** HRV, **(E)** usHRV (PSG_night subsample), and **(F)** usHRV; Subgroup health issues: **(G)** HRV, **(H)** usHRV (PSG_night subsample), and **(I)** usHRV. Transparent colored areas indicating the 95-% confidence interval.

Notes: PSG-HRV: HRV derived from entire polysomnography [5-11h]; usHRV: ultrashort HRV estimated from 10-second ECG; lnRMSSD: natural logarithm of root mean square successive difference (RMSSD) between heart beats; usHRV (PSG_night subsample): usHRV matching the polysomnography_night subsample.

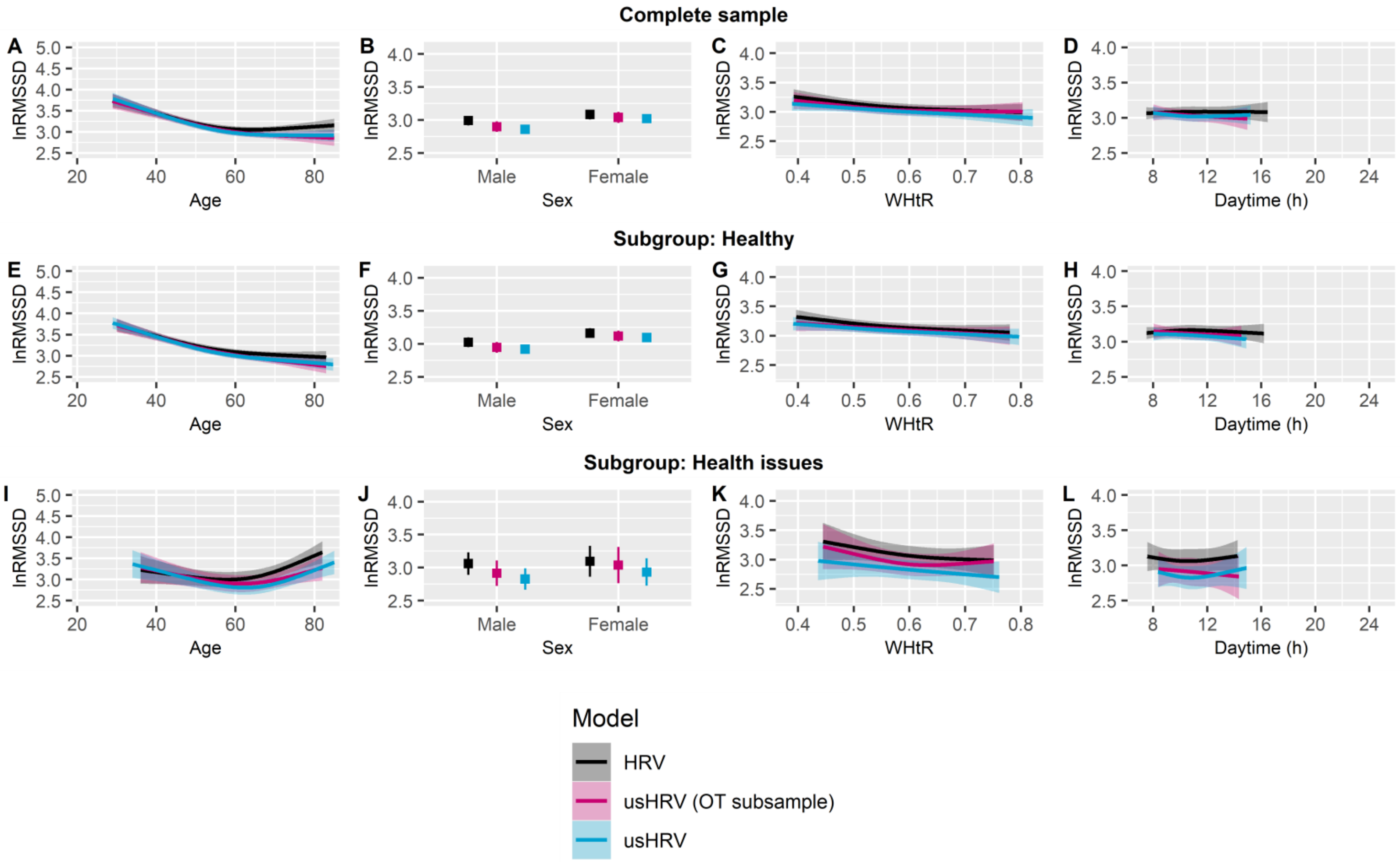

Supplementary Figure S8. Predicted lnRMSSD as a function of Age, Sex and WHtR adjusted for daytime. RMSSD was log-transformed, and the plotted values were predicted after adjustment for covariates. Complete sample – association of lnRMSSD with **(A)** age, **(B)** sex, **(C)** WHtR, and daytime **(D)**. Healthy subgroup – association of lnRMSSD with **(E)** age, **(F)** sex, **(G)** WHtR, and **(H)** daytime. Subgroup with health issues – association of lnRMSSD with **(I)** age, **(J)** sex, **(K)** WHtR, and **(L)** daytime. Transparent colored areas indicating the 95-% confidence interval.

Notes: HRV: HRV derived from rest before probing orthostatic reaction [5min]; usHRV: ultrashort HRV estimated from 10-second ECG; daytime: Daytime in hours when the ECG-recording was started; lnRMSSD: natural logarithm of root mean square successive difference (RMSSD) between heart beats; UsHRV (OT subsample): usHRV matching the orthostatic test subsample.

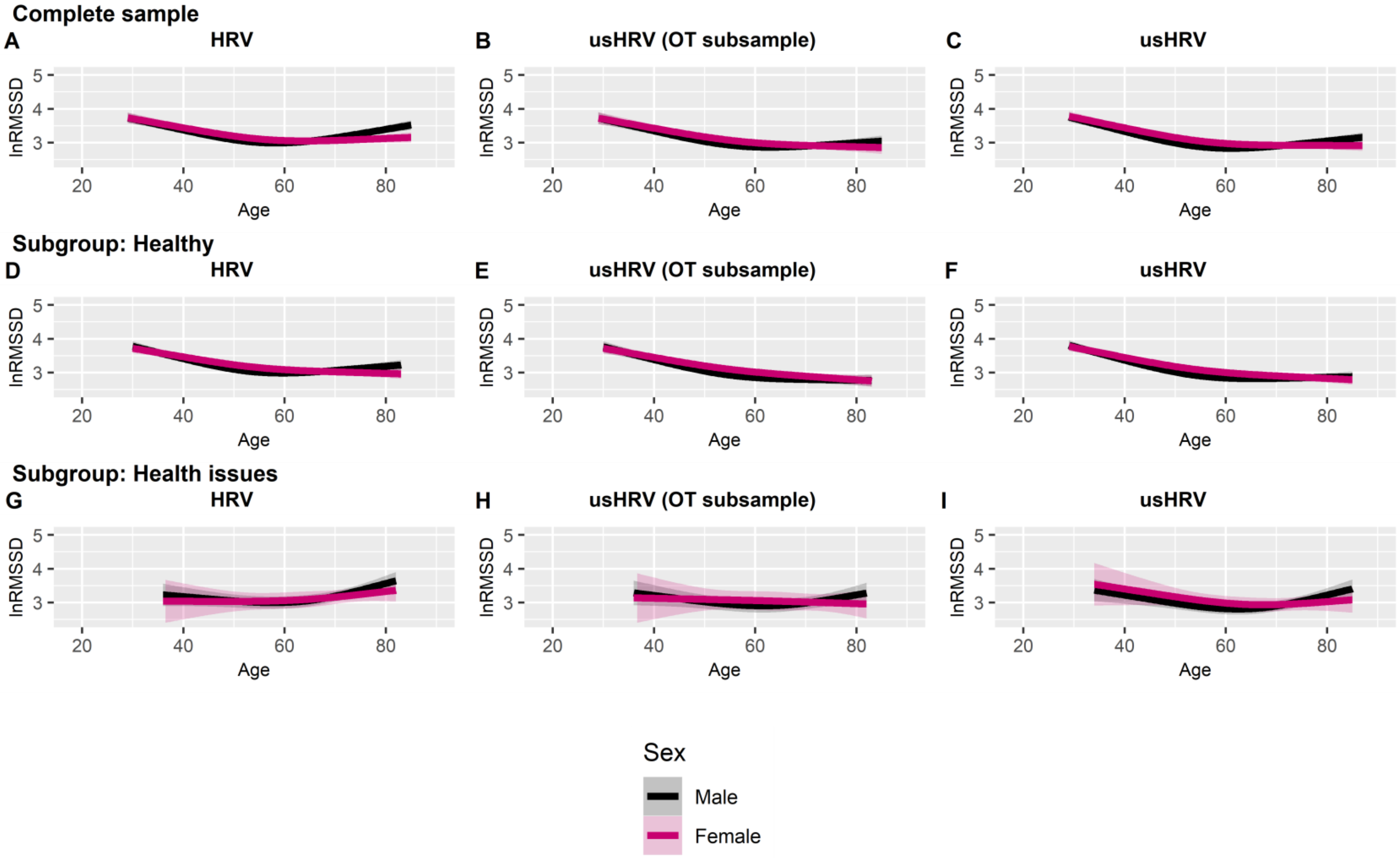

Supplementary Figure S9. Predicted lnRMSSD and the interaction of Age and Sex, adjusted for WHtR and daytime. Plotted values were predicted after adjustment for covariates. Association between predicted lnRMSSD and the interaction of Age and Sex in SHIP-TREND-1 across HRV and usHRV. RMSSD was log-transformed, and the plotted values were predicted after adjustment for covariates. Complete sample: **(A)** HRV, **(B)** usHRV (OT subsample), and **(C)** usHRV; Subgroup healthy: **(D)** HRV, **(E)** usHRV, (OT subsample), and **(F)** usHRV; Subgroup health issues: **(G)** HRV, **(H)** usHRV (OT subsample), and **(I)** usHRV. Transparent colored areas indicating the 95-% confidence interval.

Notes: HRV: HRV derived from rest before probing orthostatic reaction [5min]; usHRV: ultrashort HRV estimated from 10-second ECG; lnRMSSD: natural logarithm of root mean square successive difference (RMSSD) between heart beats; usHRV (OT subsample): usHRV matching the orthostatic test subsample.

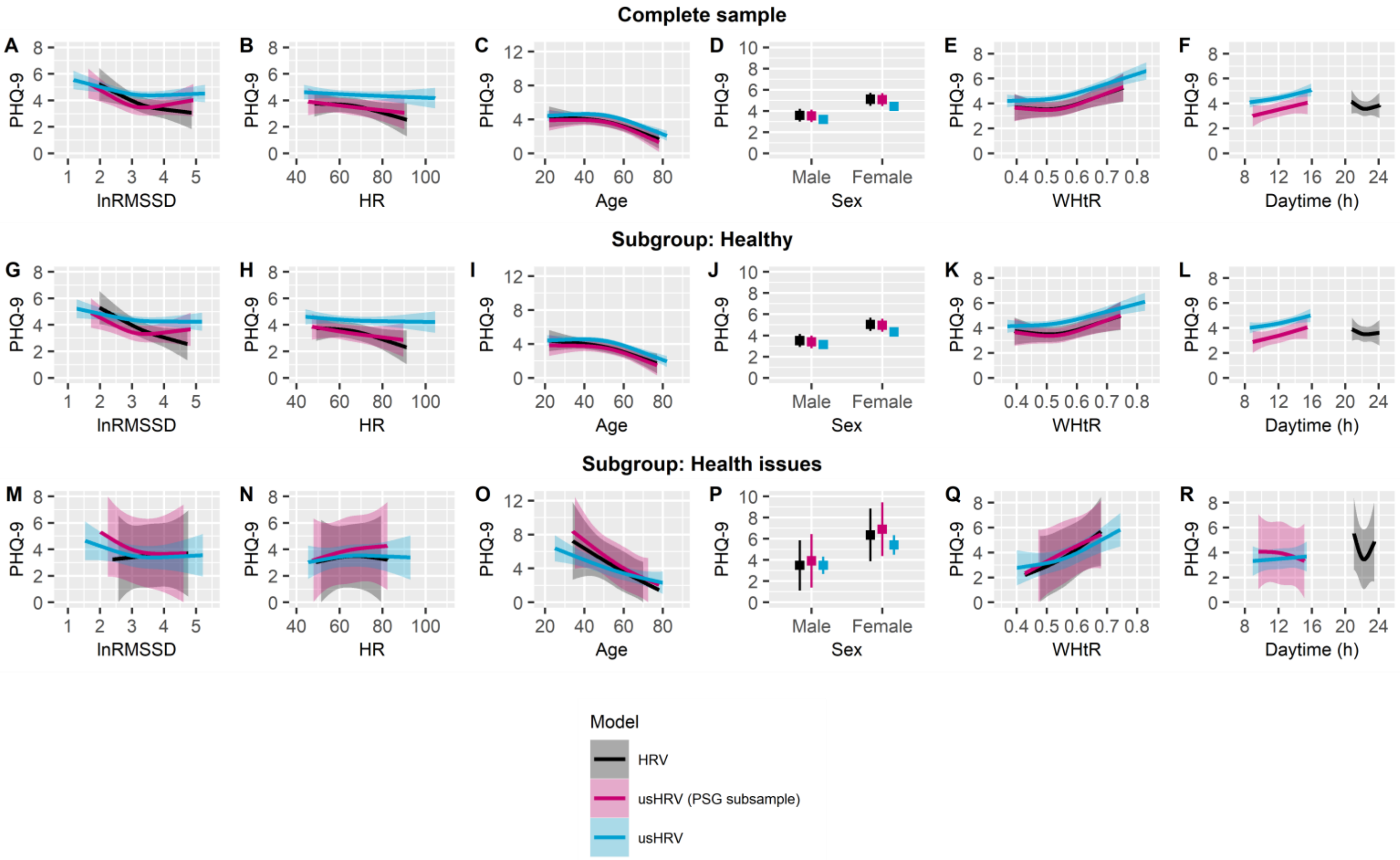

Supplementary Figure S10. Predicted PHQ-9 sum score as a function of lnRMSSD, HR, Age, Sex, WHtR and daytime. RMSSD was log-transformed, and the plotted values were predicted after adjustment for covariates. Complete sample – association of PHQ-9 sum score with **(A)** lnRMSSD **(B)** HR, **(C)** age, **(D)** sex, **(E)** WHtR, and **(F)** daytime. Healthy subgroup – association of PHQ-9 sum score with **(G)** lnRMSSD **(H)** HR, **(I)** age, **(J)** sex, **(K)** WHtR, and **(L)** daytime. Subgroup with health issues – association of PHQ-9 sum score with **(M)** lnRMSSD, **(N)** HR, **(O)** age, **(P)** sex, **(Q)** WHtR, and **(R)** daytime. Transparent colored areas indicating the 95-% confidence interval.

Notes: HRV: HRV derived from polysomnography before falling asleep [5min]; usHRV: RMSSD derived from 10-second ECG; PHQ-9: Depression module of the Patient Health Questionnaire; HR: Heartrate; daytime: Daytime in hours when the ECG-recording was started; lnRMSSD: natural logarithm of root mean square successive difference (RMSSD) between heart beats; usHRV (PSG subsample): usHRV matching the polysomnography subsample; usHRV (PSG subsample): usHRV matching the polysomnography subsample.

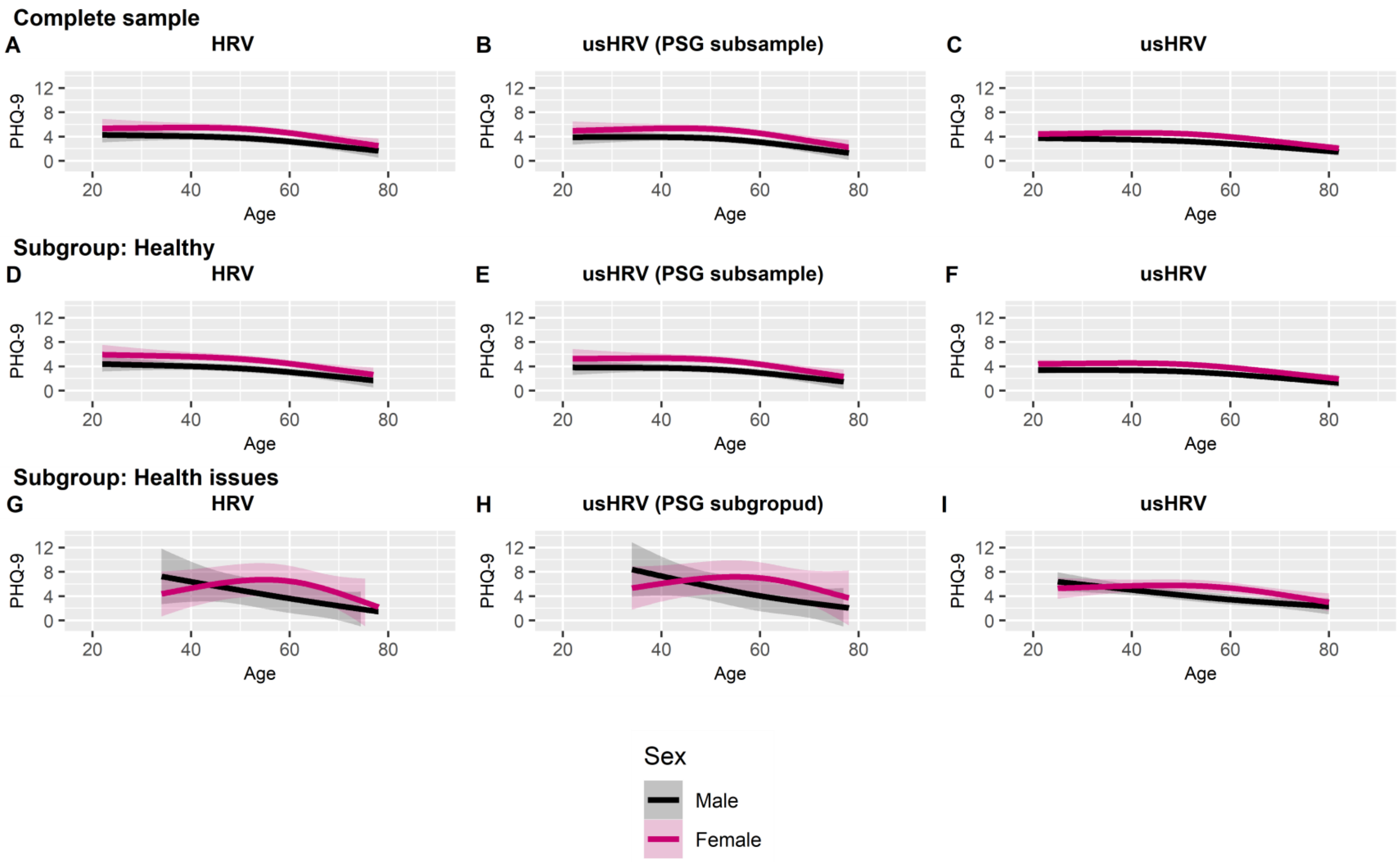

Supplementary Figure S11. Predicted PHQ-9 sum score and the interaction of Age and Sex, adjusted for lnRMSSD, HR, WHtR and daytime. Plotted values were predicted after adjustment for covariates. Complete sample: **(A)** HRV, **(B)** usHRV (PSG subsample), and **(C)** usHRV; Subgroup healthy: **(D)** HRV, **(E)** usHRV (PSG subsample), and **(F)** usHRV; Subgroup health issues: **(G)** HRV, **(H)** usHRV (PSG subsample), and **(I)** usHRV. Transparent colored areas indicating the 95-% confidence interval.

Notes: HRV: HRV derived from polysomnography before falling asleep [5min]; usHRV: RMSSD from 10-second ECG; PHQ-9: Depression module of the Patient Health Questionnaire; lnRMSSD: natural logarithm of root mean square successive difference (RMSSD) between heart beats.

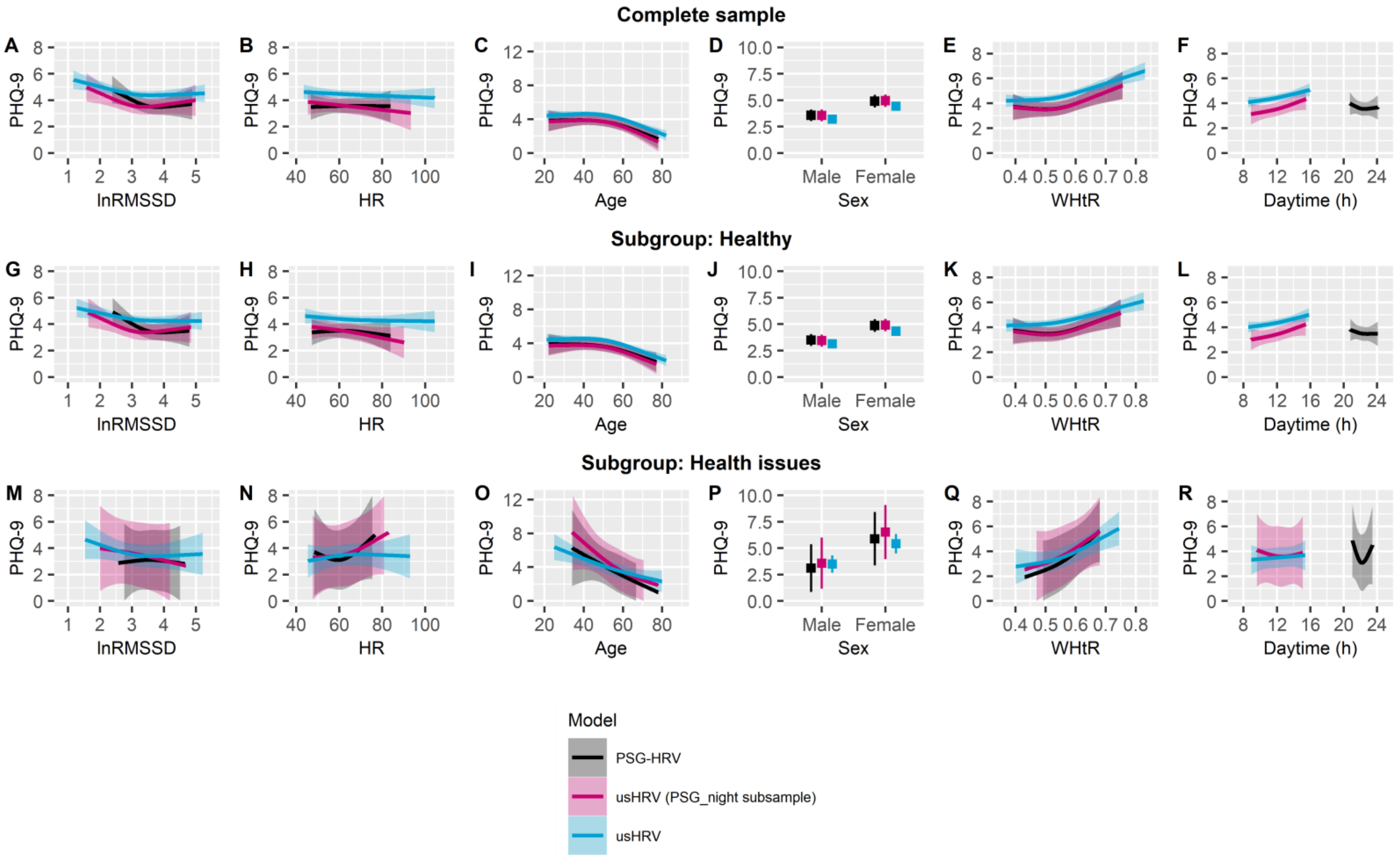

Supplementary Figure S12. Predicted PHQ-9 sum score as a function of lnRMSSD, HR, Age, Sex, WHtR and daytime. RMSSD was log-transformed, and the plotted values were predicted after adjustment for covariates. Complete sample – association of PHQ-9 sum score with **(A)** lnRMSSD, **(B)** HR, **(C)** age, **(D)** sex, **(E)** WHtR, and **(F)** daytime. Healthy subgroup – association of PHQ-9 sum score with **(G)** lnRMSSD, **(H)** HR, **(I)** age, **(J)** sex, **(K)** WHtR, and **(L)** daytime. Subgroup with health issues – association of PHQ-9 sum score with **(M)** lnRMSSD, **(N)** HR, **(O)** age, **(P)** sex, **(Q)** WHtR, and **(R)** daytime. Transparent colored areas indicating the 95-% confidence interval.

Notes: PSG-HRV: HRV derived from entire polysomnography [5-11h]; usHRV: RMSSD from 10-second ECG; PHQ-9: Depression module of the Patient Health Questionnaire; HR: Heartrate; daytime: Daytime in hours when the ECG-recording was started; lnRMSSD: natural logarithm of root mean square successive difference (RMSSD) between heart beats; usHRV (PSG_night subsample): usHRV matching the polysomnography_night subsample.

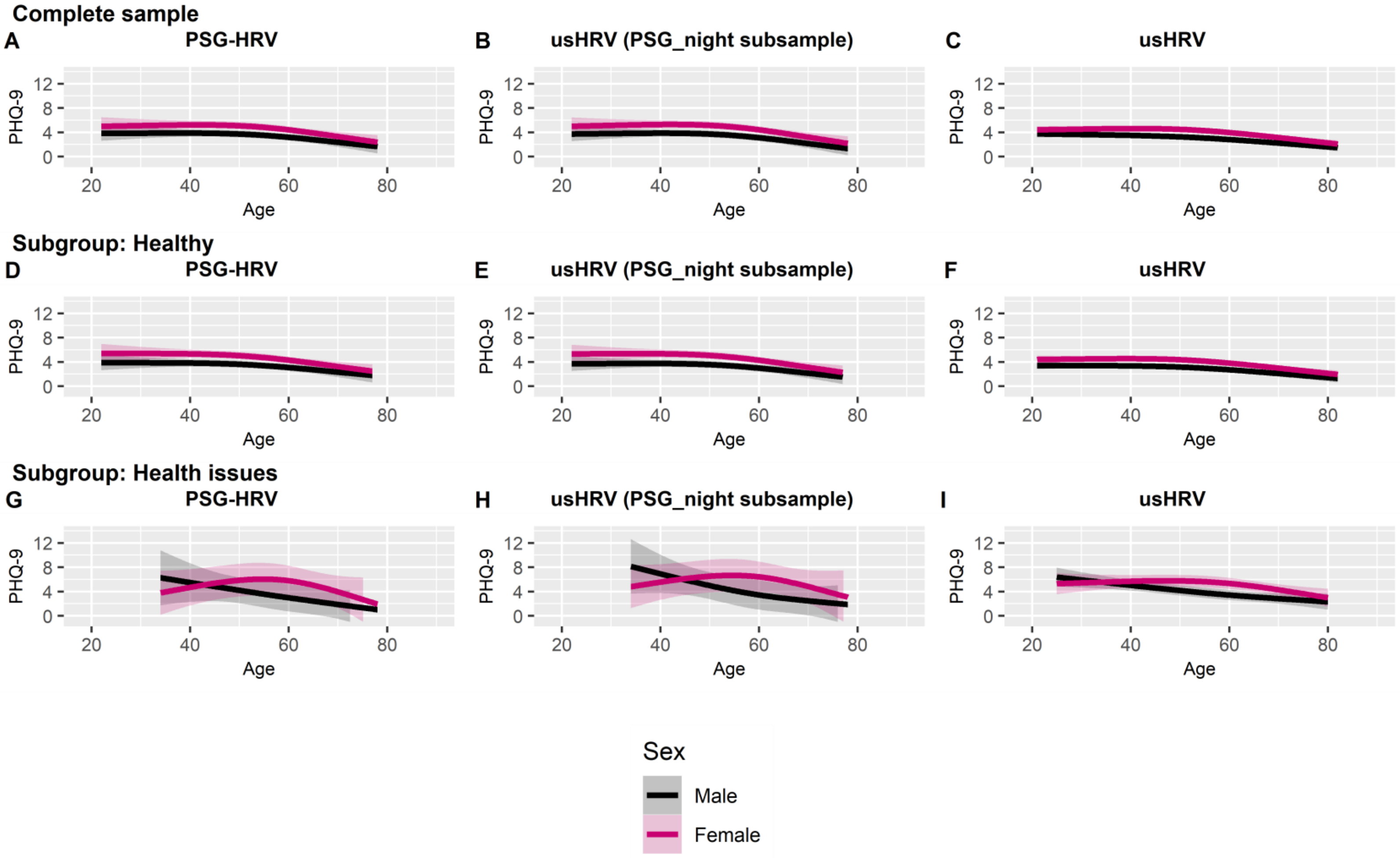

Supplementary Figure S13. Predicted PHQ-9 sum score and the interaction of Age and Sex, adjusted for lnRMSSD, HR, WHtR and daytime. Plotted values were predicted after adjustment for covariates. Complete sample: **(A)** HRV, **(B)** usHRV (PSG_night subsample), and **(C)** usHRV; Subgroup healthy: **(D)** HRV, **(E)** usHRV (PSG subsample), and **(F)** usHRV; Subgroup health issues: **(G)** HRV, **(H)** usHRV (PSG subsample), and **(I)** usHRV. Transparent colored areas indicating the 95-% confidence interval.

Notes: PSG-HRV: HRV derived from entire polysomnography [5-11h]; usHRV: RMSSD from 10-second ECG; PHQ-9: Depression module of the Patient Health Questionnaire; lnRMSSD: natural logarithm of root mean square successive difference (RMSSD) between heart beats; usHRV (PSG_night subsample): usHRV matching the polysomnography_night subsample.

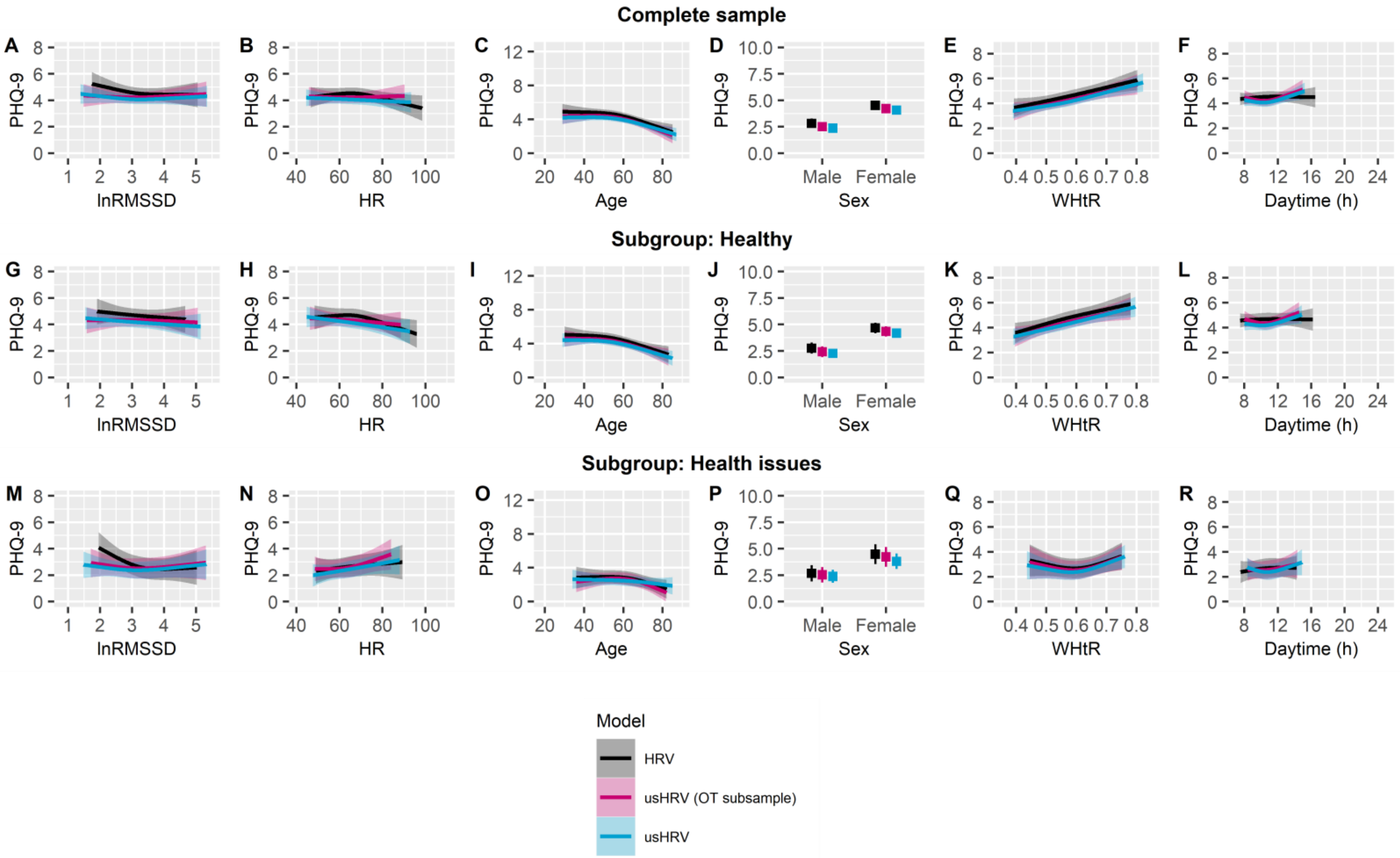

Supplementary Figure S14. Predicted PHQ-9 sum score as a function of lnRMSSD, HR, Age, Sex, WHtR and daytime. RMSSD was log-transformed, and the plotted values were predicted after adjustment for covariates. Complete sample – association of PHQ-9 sum score with **(A)** lnRMSSD, **(B)** HR, **(C)** age, **(D)** sex, **(E)** WHtR, and **(F)** daytime. Healthy subgroup – association of PHQ-9 sum score with **(G)** lnRMSSD, **(H)** HR, **(I)** age, **(J)** sex, **(K)** WHtR, and **(L)** daytime. Subgroup with health issues – association of PHQ-9 sum score with **(M)** lnRMSSD, **(N)** HR, **(O)** age, **(P)** sex, **(Q)** WHtR, and **(R)** daytime. Transparent colored areas indicating the 95-% confidence interval.

Notes: HRV: HRV derived from rest before probing orthostatic reaction [5min]; usHRV: RMSSD from 10-second ECG; PHQ-9: Depression module of the Patient Health Questionnaire; HR: Heartrate; daytime: Daytime in hours when the ECG-recording was started; lnRMSSD: natural logarithm of root mean square successive difference (RMSSD) between heart beats; usHRV (OT subsample): usHRV matching the orthostatic test subsample.

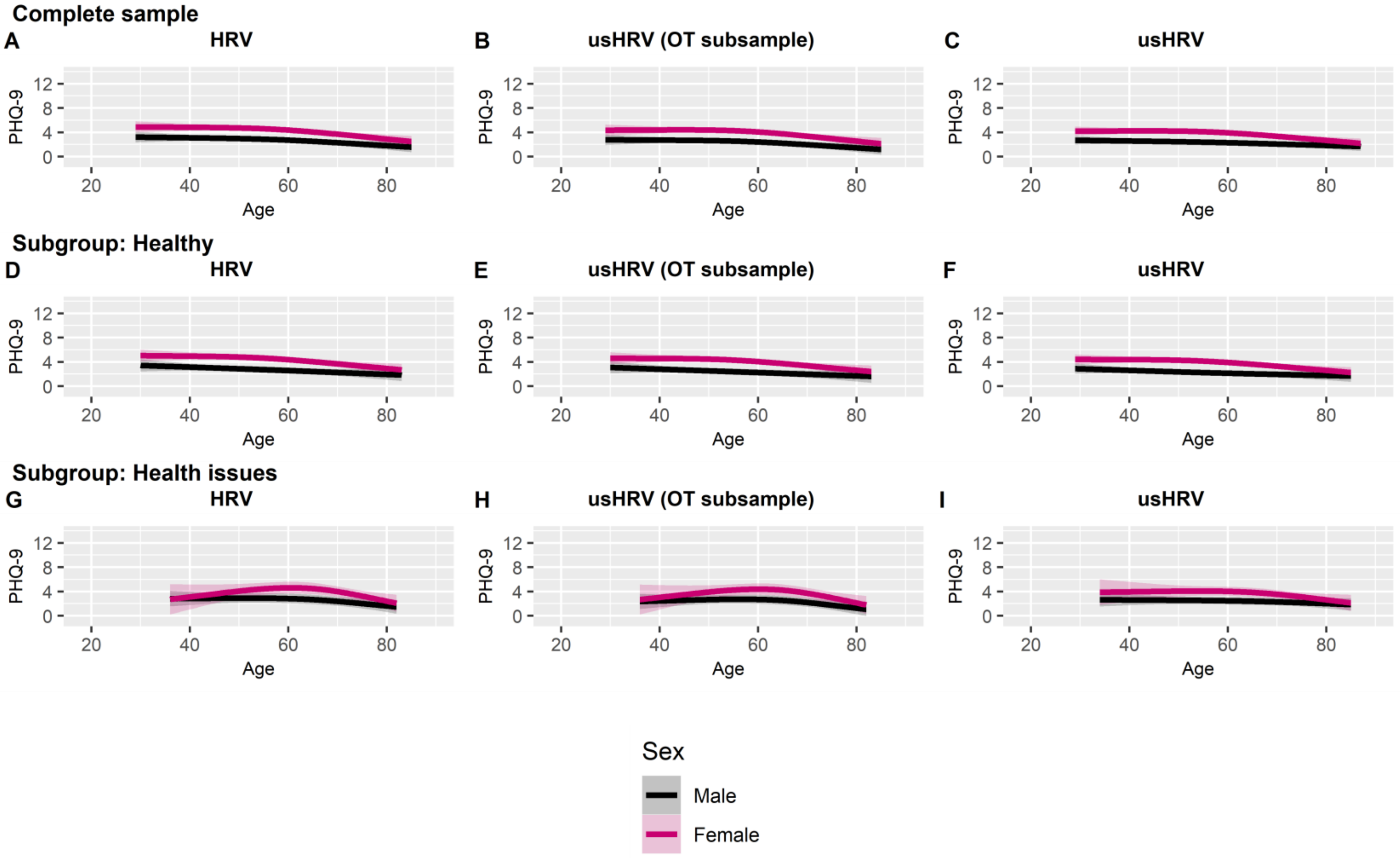

Supplementary Figure S15. Predicted PHQ-9 sum score and the interaction of Age and Sex, adjusted for lnRMSSD, HR, WHtR and daytime. Plotted values were predicted after adjustment for covariates. Complete sample: **(A)** HRV, **(B)** usHRV (OT subsample), and **(C)** usHRV; Subgroup healthy: **(D)** HRV, **(E)** usHRV (OT subsample), and **(F)** usHRV; Subgroup health issues: **(G)** HRV, **(H)** usHRV (OT subsample), and **(I)** usHRV. Transparent colored areas indicating the 95-% confidence interval.

Notes: HRV: HRV derived from rest before probing orthostatic reaction [5min]; usHRV: RMSSD from 10-second ECG; PHQ-9: Depression module of the Patient Health Questionnaire; HR: Heartrate; daytime: Daytime in hours when the ECG-recording was started; lnRMSSD: natural logarithm of root mean square successive difference (RMSSD) between heart beats; usHRV (OT subsample): usHRV matching the orthostatic test subsample.

2.2 Supplementary Tables

Table S1. Sample characteristics

Wave	SHIP-TREND-0									SHIP-TREND-1					
Sample	Polysomnography subsample [5 min]			Polysomnography_night subsample [5-11 h]			usHRV complete sample [10 second]			Orthostatic test subsample [5 min]			usHRV complete sample [10 second]		
Subgroup	Complete	Healthy	Health issues	Complete	Healthy	Health issues	Complete	Healthy	Health issues	Complete	Healthy	Health issues	Complete	Healthy	Health issues
N	1,041	930	111	1,130	1,013	117	4,245	3,623	625	1,676	1,317	359	2,392	1,854	541
Sex, *N*(%), female	487 (46.8)	446 (48.0)	41 (36.9)	524 (46.4)	482 (47.6)	42 (35.9)	2,215 (52.2)	1,951 (53.9)	267 (42.7)	848 (50.6)	730 (55.4)	118 (32.9)	1,262 (52.8)	1,060 (57.2)	205 (37.9)
p-value			.026[b]			<.001[b]			<.001[b]			<.001[b]			<.001[b]
Age [SD]	52.69 [13.76]	51.92 [13.61]	59.17 [13.34]	52.53 [13.72]	51.79 [13.60]	58.92 [13.11]	51.46 [15.25]	50.25 [15.04]	56.78 [15.42]	56.86 [13.11]	55.16 [12.70]	62.91 [12.74]	56.76 [13.71]	54.54 [13.12]	63.93 [13.21]
p-value			<.001[a]			<.001[a]			<.001[a]			<.001[a]			<.001[a]
WHtR [SD][c]	0.54 [0.08]	0.54 [0.08]	0.55 [0.08]	0.54 [0.08]	0.54 [0.08]	0.55 [0.08]	0.54 [0.08]	0.53 [0.08]	0.55 [0.08]	0.57 [0.08]	0.56 [0.08]	0.60 [0.08]	0.56 [0.08]	0.56 [0.08]	0.60 [0.08]
p-value			.076[a]			.042[a]			<.001[a]			<.001[a]			<.001[a]
BMI [SD][d]	28.44 [4.87]	28.40 [4.87]	28.76 [4.83]	28.43 [4.86]	28.39 [4.88]	28.80 [4.72]	28.03 [5.18]	27.93 [5.20]	28.60 [5.07]	28.09 [4.87]	27.71 [4.74]	29.44 [5.11]	27.96 [4.94]	27.57 [4.86]	29.24 [4.98]
			.452			.369			.002			<.001			<.001
Weight in KG [SD][d]	83.08 [15.78]	83.00 [15.80]	83.76 [15.71]	83.13 [15.79]	82.98 [15.77]	84.46 [15.93]	80.97 [16.76]	80.62 [16.77]	83.01 [16.54]	81.85 [16.44]	80.50 [16.10]	86.67 [16.73]	81.11 [16.67]	79.78 [16.40]	85.41 [16.85]
			.632			0.344			<.001			<.001			<.001
Height in cm [SD][c]	170.84 [9.18]	170.88 [9.19]	170.51 [9.11]	170.91 [9.19]	170.90 [9.16]	171.06 [9.55]	169.82 [9.40]	169.75 [9.43]	170.22 [9.22]	170.50 [9.25]	170.22 [9.27]	171.50 [9.12]	170.08 [9.46]	169.88 [9.50]	170.70 [9.31]
			.689			.861			.240			.018			.068
HRV – RMSSD [SD]	34.67 [23.60]	33.96 [21.81]	40.65 [34.73]	37.63 [24.33]	37.08 [22.64]	42.42 [35.53]				29.08 [23.67]	27.58 [19.22]	34.39 [34.69]			
p-value			.049 [a]			.115 [a]						<.001[a]			
usHRV - RMSSD [SD]	28.47 [25.79]	27.70 [23.23]	34.93 [41.08]	28.76 [26.31]	28.08 [24.10]	34.67 [40.35]	29.51 [27.01]	29.13 [25.50]	31.68 [34.35]	28.46 [30.27]	26.83 [23.94]	34.25 [45.79]	28.67 [30.18]	27.25 [24.27]	33.38 [44.05]
p-value			.071 [a]			.086[a]			.077[a]			.003[a]			.002[a]
HR long [SD]	66.95 [9.39]	64.98 [9.29]	65.32 [10.54]	62.98 [8.04]	63.14 [7.97]	61.58 [8.58]				67.71 [9.79]	68.10 [9.54]	66.32 [10.52]			
p-value			.083[a]			.063 [a]						.003[a]			
HR 10s [SD]	64.83 [9.41]	64.98 [9.29]	63.59 [10.35]	64.79 [9.54]	64.90 [9.41]	63.89 [10.63]	66.12 [10.24]	66.43 [10.18]	64.32 [10.34]	63.80 [8.92]	64.00 [8.64]	63.09 [9.84]	64.07 [9.04]	64.25 [8.78]	63.49 [9.82]
p-value			.092[a]			.330[a]			<.001[a]			.108[a]			.101[a]
PHQ-9 [SD][e]	4.4 [3.87]	4.3 [3.73]	4.9 [4.84]	4.4 [3.85]	4.3 [3.72]	4.9 [4.85]	3.9 [3.57]	3.8 [3.47]	4.5 [4.07]	3.4 [3.4]	3.5 [3.5]	3.3 [3.01]	3.4 [3.35]	3.4 [3.44]	3.4 [3.02]
p-value			.211 [a]			.207[a]			<.001 [a]			.326[a]			.876[a]

[a] t-test; [b] Mann-Whitney-U-Test; [c] SHIP-TREND-0: two missing values in PSG and PSG_night subsample, 11 missing values in usHRV complete sample; SHIP-TREND-1: 1 missing values in OT subsample, 6 missing values in usHRV complete sample; [d] SHIP-TREND-0: 2 missing values in usHRV complete sample; SHIP-TREND-1: 1 missing value in usHRV complete sample and 7 missing values in OT subsample; [e] SHIP-TREND-0: 12 missing values in PSG subsample and PSG_night subsample, 190 missing values in usHRV complete sample; SHIP-TREND-1: 2 missing values in usHRV complete sample.

Notes: usHRV: HRV derived from 10-second ECG; SHIP-TREND0: HRV: HRV derived from PSG before falling asleep [5min]; PSG-HRV: HRV derived from longest interval of laying during PSG [5-11h]; SHIP-TREND-1: HRV derived from rest before probing orthostatic reaction [5min]; RMSSD: Root mean square of successive difference between heart beats; WHtR: Waist-to-Height-Ratio; BMI: Body mass index; HRV - RMSSD: RMSSD derived from longer ECG recording; usHRV - RMSSD: RMSSD derived from 10-second ECG; HR long: Heart rate estimated from longer ECG recording; HR 10s: Heart rate estimated from 10-second ECG; PHQ-9: Depression module of the Patient Health Questionnaire; PSG: Polysomnography; OT: Orthostatic test.

Table S2. Association of sub-segmented usHRV with HRV

	SHIP-TREND-0				SHIP-TREND-1			
			HRV ~ usHRV				HRV ~ usHRV	
10s Segment	M [SD]	r	b	p	M [SD]	r	b	p
seg_01	31.4 [28.7]	.686	.690	9.1e-147	29.0 [34.8]	.695	.782	<1.8e-308
seg_02	32.9 [33.4]	.701	.741	1.8e-182	26.4 [28.3]	.681	.747	3.73e-302
seg_03	33.1 [31.9]	.692	.724	1.5e-169	26.8 [30.2]	.694	.770	<1.8e-308
seg_04	32.1 [30.7]	.708	.748	1.1e-187	26.3 [27.1]	.784	.778	<1.8e-308
seg_05	32.0 [32.1]	.704	.733	6.9e-178	26.9 [29.2]	.666	.768	<1.8e-308
seg_06	30.8 [30.2]	.703	.727	1.6e-173	27.1 [28.4]	.769	.786	<1.8e-308
seg_07	33.2 [36.3]	.643	.707	6.7e-158	26.9 [28.5]	.759	.779	<1.8e-308
seg_08	32.3 [33.0]	.653	.717	2.2e-163	26.6 [27.3]	.744	.771	<1.8e-308
seg_09	31.6 [31.7]	.699	.711	3.0e-160	26.7 [30.7]	.593	.772	<1.8e-308
seg_10	32.4 [32.8]	.655	.704	2.5e-154	26.9 [28.1]	.746	.787	<1.8e-308
seg_11	31.6 [32.7]	.699	.732	2.9e-173	26.5 [26.1]	.747	.787	<1.8e-308
seg_12	31.8 [33.7]	.736	.739	4.1e-178	26.4 [26.2]	.762	.775	<1.8e-308
seg_13	30.7 [32.5]	.732	.719	5.5e-167	27.0 [27.7]	.717	.786	<1.8e-308
seg_14	32.3 [33.1]	.678	.716	2.8e-161	26.6 [27.5]	.716	.774	<1.8e-308
seg_15	30.8 [29.7]	.676	.708	1.4e-157	27.0 [28.7]	.769	.787	<1.8e-308
seg_16	31.2 [33.2]	.739	.734	7.5e-174	26.8 [27.7]	.757	.795	<1.8e-308
seg_17	30.6 [30.2]	.743	.739	8.5e-181	26.5 [27.0]	.731	.792	<1.8e-308
seg_18	30.7 [29.4]	.725	.750	1.8e-186	27.1 [27.2]	.746	.800	<1.8e-308
seg_19	30.7 [31.5]	.679	.743	8.9e-181	26.5 [26.2]	.749	.811	<1.8e-308
seg_20	31.2 [29.5]	.725	.757	3.6e-190	26.4 [27.1]	.758	.807	<1.8e-308
seg_21	33.0 [34.1]	.731	.770	4.7e-202	26.6 [27.2]	.734	.782	<1.8e-308
seg_22	31.9 [32.4]	.709	.746	7.1e-182	27.8 [32.2]	.773	.801	<1.8e-308
seg_23	30.7 [30.8]	.749	.756	2.7e-190	26.8 [30.1]	.688	.792	<1.8e-308
seg_24	29.9 [30.3]	.749	.756	1.2e-193	27.4 [34.5]	.673	.796	<1.8e-308
seg_25	32.2 [37.5]	.727	.758	2.4e-192	26.1 [25.5]	.781	.793	<1.8e-308
seg_26	31.1 [33.0]	.725	.770	2.1e-201	26.8 [26.6]	.789	.810	<1.8e-308
seg_27	31.6 [34.3]	.702	.769	1.6e-201	26.6 [25.4]	.748	.779	<1.8e-308
seg_28	30.3 [32.0]	.734	.757	1.9e-189	27.6 [33.4]	.574	.750	3.08e-303
seg_29	32.0 [33.7]	.750	.790	1.3e-216	26.5 [26.9]	.711	.765	<1.8e-308
seg_30	30.4 [31.4]	.750	.762	1.3e-192	26.6 [29.7]	.699	.749	6.3e-303
mean [SD]	31.6 [0.9]	.710 [0.03]			26.8 [0.6]	.725 [0.1]		
range		.643 -.750				.574 - .789		

Notes: 10s Segment: segment extracted from 5-minute ECG segment; usHRV: HRV derived from 10-second ECG segment extracted from 5-minute ECG segment; SHIP-TREND-0: HRV derived from polysomnography before falling asleep [5min]; SHIP-TREND-1: HRV derived from rest. before probing orthostatic reaction [5min].

Table S3. Correlation and bias between HRV and usHRV

Wave		SHIP-TREND–0						SHIP-TREND-1		
Sample		Polysomnography subsample [5 min]			Polysomnography_night subsample [5-11 h]			Orthostatic test subsample [5 min]		
Subgroup		Complete	Healthy	Health issues	Complete	Healthy	Health issues	Complete	Healthy	Health issues
N		1,041	930	111	1,130	1,013	117	1,676	1,317	359
Difference in days	Median	8	8	11	8	8	11	27	27	26
	Range	0 - 1214	0 - 1214	0 - 805	0 - 1214	0 - 1214	0 - 805	0 - 860	0 - 860	0 - 808
Recording start time - HRV	Median	22:32	22:32	22:29	22:05	22:05	22:04	10:20	10:20	10:23
	Range	19:57 – 1:23	20:42 – 1:23	19:57 – 00:17	19:34 - 00:52	20:27 - 00:52	19:34 – 00:01	6:37 – 17:15	6:40 - 17:15	6:37 – 16:59
Recording start time - usHRV	Median	12:07	12:02	12:33	12:04	12:01	12:33	10:25	10:23	10:27
	Range	8:35 – 17:30	8:35 – 17:30	8:57 – 15:35	8:35 – 17:30	8:35 – 17:30	8:57 – 15:35	7:47 – 15:55	7:47 – 15:45	7:49 – 15:55
Correlation with usHRV	*r*	.597	.561	.700	.574	.517	.752	.616	.531	.694
	95% CI	.56 - .63	.54 - .60	.58 - .78	.53 - .61	.47 - .56	.60 - .78	.59 - .64	.49 - .57	.64 - .74
	p	1.4e-101	4.4e-78	2.1e-17	7.7e-100	1.9e-70	1.6e-22	1.8e-175	9.5e-97	5.9e-53
Bland-Altman	Bias	0.268	0.267	0.275	0.370	0.372	0.353	0.102	0.088	0.151
	95%-LoA	-0.93 to 1.47	-0.89 to 1.43	-1.21 to 1.76	-0.80 to 1.54	-0.78 to 1.53	-0.97 to 1.67	-1.09 to 1.30	-1.04 to 1.21	-1.26 to 1.57

Notes: usHRV: HRV derived from 10-second ECG; SHIP-TREND-0: HRV derived from polysomnography before falling asleep [5min]; PSG-HRV: HRV derived from longest interval of laying during polysomnography [5-11h]; SHIP-TREND-1: HRV derived from rest before probing orthostatic reaction [5min]; CI: Confidence interval; LoA: Limits of agreement.

Table S4: Association of HRV with usHRV

Wave			SHIP-TREND-0						SHIP-TREND-1		
Sample			Polysomnography subsample [5min]			Polysomnography_night subsample [5min]			Orthostatic test subsample [5 min]		
			HRV [5 min]			PSG-HRV [5-11 h]			HRV [5 min]		
Regression Model	Estimate		Complete	Healthy	Health issues	Complete	Healthy	Health issues	Complete	Healthy	Health issues
HRV ~ usHRV		N	1,041	930	111	1,130	1,013	117	1,676	1,317	359
	usHRV	β	.56	.56	.55	.56	.55	.66	.59	.57	.63
		p	5.3e-86	1.2e-77	5.8e-10	2.1e-95	4.9e-80	1.1e-15	1.3e-158	7.0e-116	5.4e-41
		$R\text{p}^2$	.31	.31	.30	.32	.30	.43	.35	.33	.40
		Adj. $R\text{p}^2$	.31	.31	.29	.32	.30	.42	.35	.33	.39
HRV ~ usHRV + covariates		N	1,039	928	111	1,128	1,011	117	1,675	1,316	359
	usHRV	β	.42	.40	.50	.47	.43	.69	.50	.42	.56
		p	9.7e-52	3.3e-41	3.5e-08	3.1e-67	1.0e-49	4.8e-15	1.8e-106	7.4e-61	1.3e-30
		$R\text{p}^2$	.54	.55	.54	.54	.55	.60	.50	.53	.51
		Adj. $R\text{p}^2$	.54	.55	.47	.54	.54	.54	.50	.53	.48

Notes: SHIP-TREND-0: HRV derived from polysomnography before falling asleep [5min]; PSG-HRV: HRV derived from longest interval of laying during polysomnography [5-11h]; SHIP-TREND-1: HRV derived from rest before probing orthostatic reaction [5min]. Covariates: number of valid RR intervals of 10-second ECG, Heart rate estimated from 10-second ECG, Heart rate estimated from longer ECG recording, Waist-to-Height-Ratio, Time difference between measurement in days, waist-to-height-ratio (WHtR), age, sex, and age X sex.

Table S5. Univariate Association of HRV with age, sex, WHtR, daytime and PHQ9

Wave	Subgroup	HRV	HRV ~ rcs(age,3)		HRV ~ Sex		HRV ~ rcs(WHtR,3)		HRV ~ rcs(Daytime,3)		PHQ9 ~ rcs(HRV,3)	
			F	***p***	***β***	***p***	***F***	***p***	***F***	***p***	***F***	***p***
SHIP-TREND-0	Complete	HRV	67.3	3.3-28	.09	.155	55.8	9.8e-24	7.0	9.5e-04	2.1	.123
		usHRV (PSG subsample)	87.6	6.8-36	.14	.022	46.4	5.0e-20	4.3	.014	1.4	.241
		usHRV	413.9	6.1-165	.18	3.4e-09	181.2	3.1e-76	11.5	1.0e-05	0.8	.449
	Healthy	HRV	72.2	7.5e-30	.10	.146	59.2	6.3e-25	6.6	.001	2.3	.099
		usHRV (PSG subsample)	93.1	1.4e-37	.14	.031	50.8	1.2e-21	4.8	.009	0.9	.401
		usHRV	398.3	4.6e-157	.21	1.9e-10	188.9	9.5e-79	10.7	2.4e-05	0.1	.866
	Health issues	HRV	2.8	.064	.09	.650	3.0	.056	0.7	.508	0.2	.851
		usHRV (PSG subsample)	3.9	.023	.18	.350	0.9	.400	0.8	.468	0.6	.535
		usHRV	34.17	8.3e-15	.03	.705	7.1	.001	1.2	.303	0.6	.538
SHIP-TREND-0	Complete	PSG-HRV	101.1	4.2e-41	-.11	.059	29.7	2.8e-13	0.8	.441	2.4	.087
		usHRV (PSG_night subsample)	91.6	1.4e-37	.12	.039	46.8	3.1e-20	5.2	.006	0.9	.426
		usHRV	413.9	6.1e-165	.18	3.4e-09	181.2	3.1e-76	11.5	1.0e-05	0.8	.449
	Healthy	PSG-HRV	108.2	2.7e-43	-.11	.061	33.8	6e-15	0.7	.489	2.3	.099
		usHRV (PSG_night subsample)	95.6	9.7e-39	.13	.043	49.8	2.3e-21	6.2	.002	0.8	.445
		usHRV	398.3	4.6e-157	.21	1.9e-10	188.9	9.5e-79	10.7	2.4e-05	0.1	.866
	Health issues	PSG-HRV	3.1	.047	-.04	.841	0.35	.710	0.5	.609	0.4	.685
		usHRV (PSG_night subsample)	4.5	.013	.13	.502	1.2	.310	0.6	.568	0.5	.631
		usHRV	34.2	8.3e-15	.08	.705	7.1	.001	1.2	.303	0.6	.538
SHIP-TREND-1	Complete	HRV	87.1	1.0e-36	.06	.228	33.7	4.5e-15	1.0	.373	1.0	.381
		usHRV (OT subsample)	99.4	1.6e-41	.14	4.7e-03	38.3	5.6e-17	2.2	.108	0.9	.410
		usHRV	156.0	2.0e-64	.15	3.2e-04	58.8	1.1e-25	1.6	.193	.01	.874
	Healthy	HRV	106.3	1.6e-43	.12	.030	43.1	7.3e-19	1.0	.375	0.8	.433
		usHRV (OT subsample)	130.6	1.8e-52	.19	5.0e-04	41.6	2.9e-18	2.1	.123	1.7	.176
		usHRV	191.5	2.6e-76	.18	9.7e-05	62.9	3.8e-27	3.1	.044	0.6	.548
	Health issues	HRV	8.1	3.5e-04	-.06	.586	1.7	.180	0.1	.914	6.8	.001
		usHRV (OT subsample)	3.5	.032	.001	.996	3.1	.046	0.3	.745	0.3	.747
		usHRV	10.6	3.0e-05	.04	.684	4.0	.019	0.4	.642	0.8	.566

Notes usHRV: HRV derived from 10-second ECG; SHIP-TREND-0: HRV derived from polysomnography before falling asleep [5min]; PSG-HRV: HRV derived from longest interval of laying during polysomnography [5-11h]; SHIP-TREND-1: HRV derived from rest. before probing orthostatic reaction [5min]. WHtR: Waist-to-Height-Ratio; usHRV (PSG subsample): usHRV matching the polysomnography subsample [5 min]; usHRV (PSG_night subsample): usHRV matching the polysomnography_night subsample [5-11h]; usHRV (OT subsample): usHRV matching the orthostatic test subsample; rcs: restricted cubic spline with 3 knots.

Table S6. Multivariate associations of HRV and usHRV with Age, Sex, Age X Sex, WHtR and Daytime

Wave	Subgroup	HRV	N	Age		Sex		Age X Sex		WHtR		Daytime				Δ Model-fit	
				F	*p*	*β*	*p*	*F*	*p*	*F*	*p*	*F*	*p*	R^2	R_{adj}^2	*z*	*p*
SHIP-TREND-0	Complete	HRV	1,039	30.4	1.5e-13	.098	.476	0.3	.713	17.4	3.8e-08	3.2	.042	.155	.147		
		usHRV (PSG subsample)	1,039	50.0	1.9e-21	-.017	.902	1.6	.200	8.1	3.4e-04	2.2	.112	.172	.165	0.81	.419
		usHRV	4,234	237.0	2.1e-98	.180	.009	0.0	.850	11.2	1.4e-05	2.1	.128	.176	.174		
	Healthy	HRV	928	31.7	4.8e-14	-.025	.861	2.5	.086	15.7	1.9e-07	2.3	.106	.180	.172		
		usHRV (PSG subsample)	928	52.7	2.1e-22	-.0004	.998	2.0	.133	8.3	2.6e-04	2.8	.061	.203	.195	0.93	.353
		usHRV	3,614	222.0	1.8e-91	.232	.001	0.3	.745	15.0	3.2e-07	2.2	.114	.199	.197		
	Health issues	HRV	111	1.9	.159	.582	.239	1.3	.272	2.8	.064	1.3	.269	.136	.059		
		usHRV (PSG subsample)	111	2.7	.075	.230	.647	1.1	.350	0.1	.868	0.5	.620	.105	.026	-0.44	.661
		usHRV	623	25.1	3.3e-11	-.079	.697	1.2	.293	0.2	.844	0.2	.832	.104	.091		
SHIP-TREND-0	Complete	PSG-HRV	1,128	64.2	4.2e-27	-.235	.073	1.5	.236	1.1	.329	0.7	.509	.159	.152		
		usHRV (PSG_night subsample)	1,128	51.2	5.5e-22	-.030	.816	1.7	.176	7.1	8.8e-04	2.3	.102	.163	.157	0.21	.831
		usHRV	4,234	237.0	2.1e-98	.180	.009	0.2	.850	11.2	1.4e-05	2.1	.128	.176	.174		
	Healthy	PSG-HRV	1,011	65.5	1.8e-27	-.315	.023	3.3	.038	1.3	.266	0.4	.662	.186	.178		
		usHRV (PSG_night subsample)	1,011	53.2	1.1e-22	-.001	.993	2.0	.143	7.0	9.8e-04	3.6	.029	.189	.182	0.17	.869
		usHRV	3,614	222.0	1.8e-91	.232	.001	0.3	.745	15.0	3.2e-07	2.2	.114	.199	.197		
	Health issues	PSG-HRV	117	3.3	.042	.160	.749	0.2	.857	0.2	.807	0.9	.421	.078	<.001		
		usHRV (PSG_night subsample)	117	3.2	.044	.153	.756	1.2	.298	0.2	.859	0.4	.702	.108	.034	0.54	.588
		usHRV	623	25.1	3.4e-11	-.079	.697	1.2	.293	0.2	.844	0.2	.832	.104	.091		
SHIP-TREND-1	Complete	HRV	1,675	59.3	1.4e-25	.269	.012	7.4	6.2e-04	6.1	.002	0.1	.872	.111	.106		
		usHRV (OT subsample)	1,675	64.2	1.4e-27	.284	.007	2.6	.075	2.8	.062	0.9	.424	.117	.112	0.44	.658
		usHRV	2,386	104.0	5.7e-44	.329	.3.2e-04	5.5	.004	4.6	.010	0.5	.594	.129	.126		
	Healthy	HRV	1,316	70.2	1.2e-29	.384	.001	5.1	.007	6.5	.002	0.4	.646	.160	.154		
		usHRV (OT subsample)	1,316	91.8	4.8e-38	.354	.003	1.5	.218	3.0	.051	0.5	.615	.181	.176	1.14	.256
		usHRV	1,849	132.0	4.2e-54	.360	1.9e-04	2.6	.075	4.4	.012	0.8	.471	.187	.183		
	Health issues	HRV	359	8.3	3.1e-04	.204	.456	0.8	.460	1.8	.171	0.2	.795	.059	.035		
		usHRV (OT subsample)	359	1.7	.179	.320	.249	1.1	.341	1.3	.264	0.2	.814	.034	.009	-1.08	.282
		usHRV	538	7.3	7.5e-04	.231	.273	1.8	.165	1.3	.283	0.4	.677	.049	.033		

Notes: usHRV: HRV derived from 10-second ECG; SHIP-TREND-0: HRV derived from polysomnography before falling asleep [5min]; PSG-HRV: HRV derived from longest interval of laying during polysomnography [5-11h]; SHIP-TREND-1: HRV derived from rest before probing orthostatic reaction [5min]. WHtR: Waist-to-Height-Ratio; usHRV (PSG subsample): usHRV matching the polysomnography subsample [5 min]; usHRV (PSG_night subsample): usHRV matching the polysomnography_night subsample [5-11h]; usHRV (OT subsample): usHRV matching the orthostatic test subsample; Δ Model-fit: statistical comparison of the correlations between actual and predicted values of HRV/PSG-HRV and the matching usHRV subsample (PSG subsample, PSG_night subsample, OT subsample), performed with cocor.

Table S7. Multivariate associations of PHQ-9 with HRV and usHRV

Wave	Subgroup	HRV	N	lnRMSSD		HR		Age		Sex		Age*Sex		Daytime		WHtR				Anova	Δ Model-fit	
				F	*p*	*F*	*p*	*F*	*p*	*β*	*p*	*F*	*p*	*F*	*p*	*F*	*p*	R^2	R_{adj}^2	*p*	*z*	*p*
SHIP-TREND-0	Complete	HRV	1,027	4.6	.010	1.8	.170	15.1	3.6e-07	.151	.099	0.3	.742	0.7	.511	4.6	.010	.068	.056			
		usHRV (PSG subsample)	1,027	4.7	.010	0.8	.435	17.0	5.4e-08	.162	.077	0.2	.784	2.6	.075	5.2	.006	.071	.059	.208	0.42	.678
		usHRV	4,044	5.3	.005	0.7	.502	42.4	5.7e-19	.121	.014	1.2	.310	8.1	3.0e-04	23.7	6.0e-11	.047	.044			
	Healthy	HRV	918	6.7	.001	2.4	.095	14.2	8.2e-07	.160	.099	0.3	.772	0.4	0.7	3.4	.033	.075	.061			
		usHRV (PSG subsample)	918	3.6	.029	1.2	.300	14.1	9.1e-07	.179	.067	0.4	.697	3.1	.047	4.2	.015	.074	.061	.057	-0.03	.974
		usHRV	3,451	3.6	.027	0.7	.511	37.0	1.3e-16	.101	.068	0.6	.552	7.5	5.8e-04	14.3	6.6e-07	.047	.044			
	Health issues	HRV	109	<0.1	.972	0.1	.949	3.2	.047	.094	.759	1.8	.168	1.2	.321	2.2	.114	.156	.041			
		usHRV (PSG subsample)	109	0.7	.488	0.2	.823	2.8	.069	-.021	.944	2.0	.145	0.1	.881	1.7	.180	.163	.049	.622	0.22	.827
		usHRV	596	1.4	.245	0.3	.756	11.6	1.2e-05	.132	.231	3.0	.053	0.2	.843	9.6	8.1e-05	.085	.065			
SHIP-TREND-0	Complete	PSG-HRV	1,116	2.9	.057	<0.1	.985	13.7	1.3e-06	.170	.055	0.3	.775	0.4	.649	5.8	.003	.063	.052			
		usHRV (PSG_night subsample)	1,116	3.3	.036	0.8	.441	17.7	2.8e-08	.169	.056	0.2	.809	3.8	.023	6.2	.002	.067	.056	.142	0.61	.543
		usHRV	4,044	5.3	.005	0.7	.502	42.4	5.7e-19	.121	.014	1.2	.310	8.1	3.0e-04	23.7	6.0e-11	.047	.044			
	Healthy	PSG-HRV	1,001	3.8	.024	0.2	.813	12.5	4.5e-06	.176	.064	0.5	.626	0.3	.753	4.5	.012	.066	.054			
		usHRV (PSG_night subsample)	1,001	3.3	.038	1.8	.161	15.0	3.8e-07	.178	.059	0.5	.598	3.7	.025	4.9	.008	.072	.060	.064	0.62	.536
		usHRV	3,451	3.6	.027	0.7	.511	37.0	1.3e-16	.101	.068	0.6	.552	7.5	5.8e-04	14.3	6.6e-07	.047	.044			
	Health issues	PSG-HRV	115	<0.1	.972	0.8	.461	2.6	.079	.150	.595	1.6	.211	1.0	.371	2.9	.062	.162	.054			
		usHRV (PSG_night subsample)	115	0.4	.694	0.8	.462	2.5	.084	.111	.692	2.2	.121	0.1	.933	2.0	.143	.155	.046	.678	-0.17	.864
		usHRV	596	1.4	.245	0.3	.756	11.6	1.2e-05	.132	.231	3.0	.053	0.2	.843	9.6	8.1e-05	.085	.065			
SHIP-TREND-1	Complete	HRV	1,675	1.7	.185	2.7	.066	14.1	8.5e-07	.013	.856	0.8	.471	0.3	.718	12.1	5.8e-06	.076	.069			
		usHRV (OT subsample)	1,675	0.2	.833	<0.1	.953	13.3	1.9e-06	.012	.862	0.7	.512	2.3	.096	11.4	1.2e-05	.075	.068	.095	-0.27	.784
		usHRV	2,385	0.7	.491	0.4	.680	11.2	1.4e-05	.047	.412	2.3	.105	3.4	.033	17.0	4.8e-08	.067	.062			
	Healthy	HRV	1,316	0.5	.593	3.7	.026	9.5	8.4e-05	-.032	.687	0.9	.409	<0.1	.915	11.1	1.7e-05	.076	.067			
		usHRV (OT subsample)	1,316	<0.1	.942	0.4	.642	8.2	2.9e-04	-.032	.693	1.0	.374	1.9	.151	10.3	3.5e-05	.074	.065	.295	-0.50	.618
		usHRV	1,849	0.7	.488	2.5	.083	9.7	6.3e-05	.008	.899	1.8	.170	2.6	.076	15.4	2.3e-07	.072	.065			
	Health issues	HRV	359	3.7	.027	0.5	.629	5.5	.004	.246	.065	1.1	.342	0.3	.730	2.1	.126	.138	.105			
		usHRV (OT subsample)	359	0.3	.709	2.1	.119	7.1	9.8e-04	.254	.059	0.6	.538	0.4	.658	2.3	.102	.124	.091	.049	-0.79	.429
		usHRV	537	0.5	.630	2.1	.122	3.0	.048	.244	.028	1.0	.383	1.0	.360	3.7	.026	.087	.065			

Notes: lnRMSSD: natural logarithm of root mean square successive difference (RMSSD) between heart beats; usHRV: HRV derived from 10-second ECG; SHIP-TREND-0: HRV derived from polysomnography before falling asleep [5min]; PSG-HRV: HRV derived from longest interval of laying during polysomnography [5-11h]; SHIP-TREND-1: HRV derived from rest. before probing orthostatic reaction [5min]. WHtR: Waist-to-Height-Ratio; HR: Heart rate; usHRV (PSG subsample): usHRV matching the polysomnography subsample [5 min]; usHRV (PSG_night subsample): usHRV matching the polysomnography_night subsample [5-11h]; usHRV (OT subsample): usHRV matching the orthostatic test subsample; Δ Model-fit: statistical comparison of the correlations between actual and predicted values of HRV/PSG-HRV and the matching usHRV subsample (PSG subsample, PSG_night subsample, OT subsample), performed with cocor.
